# Maternal nutritional status modifies heat-associated growth restriction in women with chronic malnutrition

**DOI:** 10.1093/pnasnexus/pgac309

**Published:** 2023-01-27

**Authors:** Kartik Shankar, Sumera A Ali, Meghan L Ruebel, Saleem Jessani, Sarah J Borengasser, Stephanie P Gilley, Puujee Jambal, Deaunabah N Yazza, Nicholas Weaver, Jennifer F Kemp, Jamie L Westcott, Audrey E Hendricks, Sarah Saleem, Robert L Goldenberg, K Michael Hambidge, Nancy F Krebs

**Affiliations:** Department of Pediatrics, Section of Nutrition, University of Colorado School of Medicine, Aurora, CO 80045, USA; Aga Khan University, Karachi 7480​0, Pakistan; Department of Pediatrics, Section of Nutrition, University of Colorado School of Medicine, Aurora, CO 80045, USA; USDA-ARS, Southeast Area, Arkansas Children’s Nutrition Center, Little Rock, AR 72202, USA; Aga Khan University, Karachi 7480​0, Pakistan; Department of Pediatrics, Section of Nutrition, University of Colorado School of Medicine, Aurora, CO 80045, USA; Department of Pediatrics, Section of Nutrition, University of Colorado School of Medicine, Aurora, CO 80045, USA; Department of Pediatrics, Section of Nutrition, University of Colorado School of Medicine, Aurora, CO 80045, USA; Department of Pediatrics, Section of Nutrition, University of Colorado School of Medicine, Aurora, CO 80045, USA; Department of Mathematical and Statistical Sciences, University of Colorado Denver, CO 80204, USA; Department of Pediatrics, Section of Nutrition, University of Colorado School of Medicine, Aurora, CO 80045, USA; Department of Pediatrics, Section of Nutrition, University of Colorado School of Medicine, Aurora, CO 80045, USA; Department of Mathematical and Statistical Sciences, University of Colorado Denver, CO 80204, USA; Aga Khan University, Karachi 7480​0, Pakistan; Department of Obstetrics and Gynecology, Columbia University, New York, NY 10032, USA; Department of Pediatrics, Section of Nutrition, University of Colorado School of Medicine, Aurora, CO 80045, USA; Department of Pediatrics, Section of Nutrition, University of Colorado School of Medicine, Aurora, CO 80045, USA

**Keywords:** heat stress, pregnancy, growth restriction, malnutrition, climate change

## Abstract

Rapid changes in the global climate are deepening existing health disparities from resource scarcity and malnutrition. Rising ambient temperatures represent an imminent risk to pregnant women and infants. Both maternal malnutrition and heat stress during pregnancy contribute to poor fetal growth, the leading cause of diminished child development in low-resource settings. However, studies explicitly examining interactions between these two important environmental factors are lacking. We leveraged maternal and neonatal anthropometry data from a randomized controlled trial focused on improving preconception maternal nutrition (Women First Preconception Nutrition trial) conducted in Thatta, Pakistan, where both nutritional deficits and heat stress are prevalent. Multiple linear regression of ambient temperature and neonatal anthropometry at birth (*n* = 459) showed a negative association between daily maximal temperatures in the first trimester and *Z*-scores of birth length and head circumference. Placental mRNA-sequencing and protein analysis showed transcriptomic changes in protein translation, ribosomal proteins, and mTORC1 signaling components in term placenta exposed to excessive heat in the first trimester. Targeted metabolomic analysis indicated ambient temperature associated alterations in maternal circulation with decreases in choline concentrations. Notably, negative impacts of heat on birth length were in part mitigated in women randomized to comprehensive maternal nutritional supplementation before pregnancy suggesting potential interactions between heat stress and nutritional status of the mother. Collectively, the findings bridge critical gaps in our current understanding of how maternal nutrition may provide resilience against adverse effects of heat stress in pregnancy.

Significance Statement In a resource-limited setting, exposure to excessive ambient heat in early pregnancy is associated with lower length and head circumference at birth. These changes are associated with alterations in placental gene expression and metabolite levels in the mother. Improving maternal nutritional status through supplementation early in pregnancy may promote resilience against some of the effects of heat stress.

## Introduction

A confluence of the ongoing epidemics of malnutrition and climate change is occurring at an unprecedented pace (1). Climate impacts on human health occur through a number of sectors, many closely aligned with food systems and human nutrition (2, 3). Despite this, few studies have examined interactions between environmental health effects and nutritional deficits. Emerging evidence provide strong support linking multiple climate-sensitive health outcomes (viz. heat exposure, air quality) to detrimental outcomes in pregnancy (4). Notably, existing health and health-care disparities and poor resilience to a changing environment place women and young children with nutritional deficits in resource-limited settings at particularly high risk (3, 5, 6). Thus, studies examining the nexus of nutrition and climate effects in pregnancy are acutely needed.

Extreme heat affecting human health is not only a future risk but has contemporary ongoing impacts globally. Exposure to increasing heat and extreme heat events are hallmarks of climate change (7). Higher average air temperatures lead to more frequent periods of extremely hot weather. These events are becoming more frequent and perhaps regular features in many parts of the world (8). Climate models indicate that even if the aggressive goals of limiting global average temperature increases to 1.5°C are met, the frequency and duration of extreme heat events are still likely to increase (3). Children born in 2020 are estimated to experience two to seven-fold increased risk of extreme weather events, relative to those born in 1960, underscoring the systemically greater risk to heat waves among other events (9). At current warming of 1.2°C since preindustrial levels, extreme heat events have become increasingly common, with projections suggesting high likelihoods of record-breaking week-long heat events in the next 60 y under high-emission scenarios (10). Recent unprecedented heat waves in March to May of 2022 in the South Asian subcontinent are consistent with such projections and attributable to climate change based on work from the World Weather Attribution Initiative. Over 1 billion people in India and Pakistan were potentially exposed to ambient temperatures over >40°C over several weeks, with recorded temperatures reaching 50°C (120°F) in Jacobabad, Pakistan.

Effects of extreme heat also ripple through other sectors such as decreasing crop yields and nutrient quality of staple foods, affecting food systems, and increasing the risk of fires impacting air quality. Thus, a large proportion of the world’s population is at immediate risk for both acute and downstream effects of heat related health effects (4). Typically, children under the age of 5 y, adults > 65, and those with pre-existing health conditions are considered most vulnerable to the effects of high temperatures. However, accumulating evidence suggests that maternal and child health outcomes are adversely affected by heat exposure (4, 6, 11). Moreover, in animal models prolonged heat exposure in pregnancy results in decreased food intake, increased fetal growth retardation, placental insufficiency, impaired gut barrier function, and inflammation (12–14). However, only a few studies to date have examined the influence of heat stress (HS) on placental function and fetal health in humans (15–17).

**Fig. 1.**
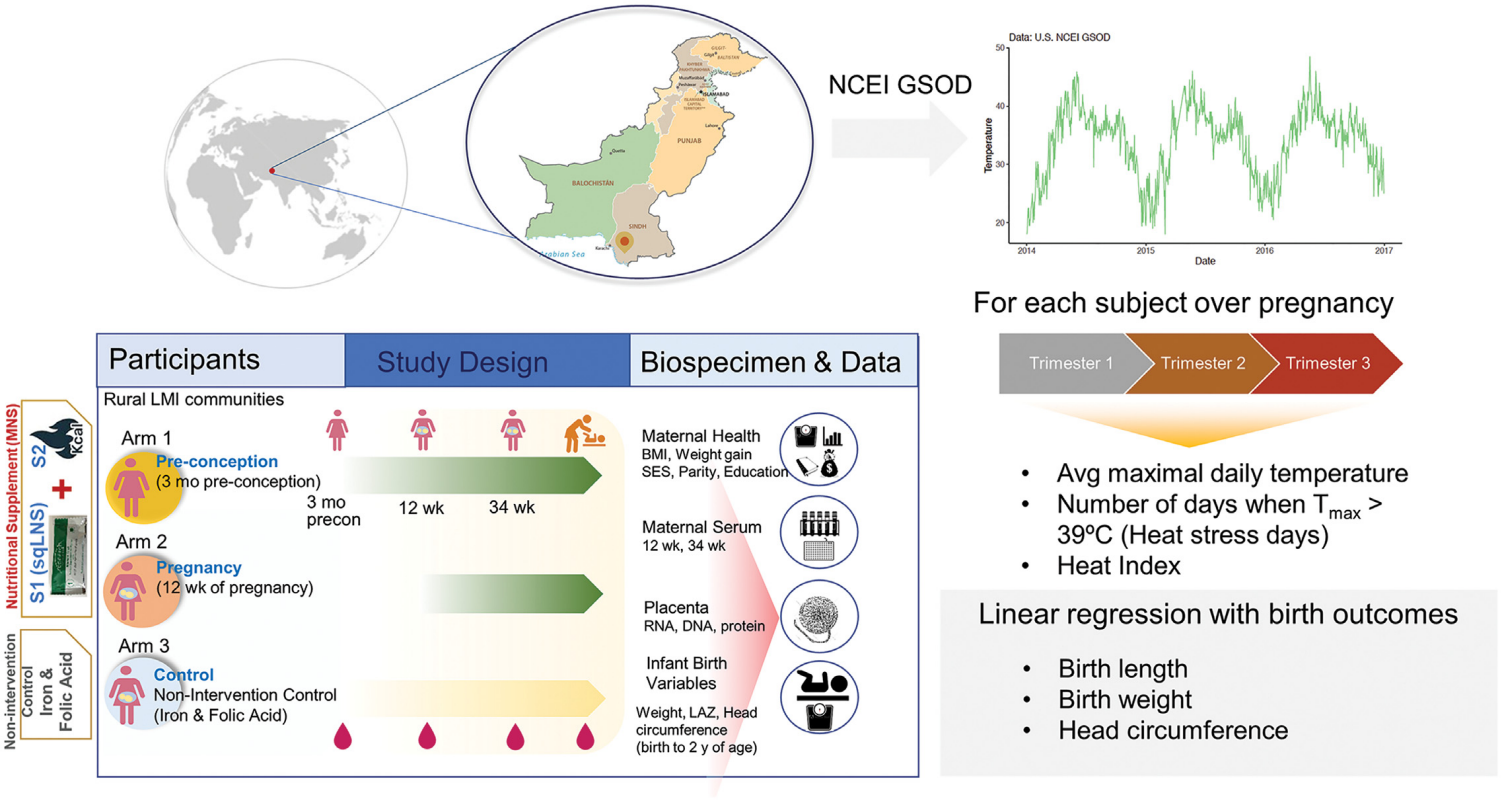
Schematic showing study design and outcomes. (A) Geographical region where the Women First Study was conducted in Thatta, Sindh (red marker, in southeastern Pakistan). (B) Exemplar plots of daily maximum air temperatures retrieved for the study period from local weather stations. (C) Design of the Women First Preconception Nutrition trial that included randomization of nonpregnant women to one of three arms. Arms 1 and 2 received a comprehensive nutritional supplementation, which included a small-quantity lipid-based nutritional supplement and additional calories from either minimum 3 months preconception or from 12 wk of pregnancy until birth. Women in Arm 3 served as unsupplemented controls. (D) Analysis strategy describing linear regression analysis of heat exposure variables derived from maximal daily temperature against infant birth outcome variables (gestational-age adjusted *Z*-scores of birth weight, birth length, and head circumference).

In the present study, we examined associations between ambient temperature during pregnancy trimesters and birth anthropometry of infants in a resource-limited setting. To this end, we leveraged data and biospecimens collected as part of a randomized controlled efficacy trial of comprehensive nutritional supplementation (Women First Maternal Preconception Nutrition Trial (WF, clinicaltrials.gov NCT01883193) (18) along with air temperature and other environmental data from meteorological and satellite-based records (Fig. 1). This analysis focused on one study site based in Thatta (Sindh province), Pakistan. Thatta is situated west of the river Indus and is a semi-arid region. Thatta has a subtropical climate and experiences very hot summers and cold winters. Temperatures frequently rise above 46°C (115°F) between May and August. Annual precipitation averages about 7 inches (180 mm), falling mainly during late summer. Combined with little access to air-conditioning or other measures of heat mitigation, exposure to high temperatures is likely uniform. Women in this area of rural Pakistan are susceptible to both poor nutrition and seasonal high ambient temperatures (19). High prevalence of hypozincemia, anemia, and low to moderate dietary diversity is common among women of child-bearing age (20). Infants in this region are also prone to low-birth weights or small for gestational-age and have high likelihood of stunting (21). Thus, this site provides a unique opportunity to study the double burden of nutrition and heat-related exposures on maternal and child outcomes. Specifically, we examined if ambient temperature and excessive heat exposure (temperatures > 39°C) impact gestational age-adjusted birth weight, length, and head circumference measures. Second, we investigated whether ambient temperature was associated with mRNA and protein expression in the term placenta linking temperature to fetal growth. Using a combination of mRNA-sequencing of 126 placenta samples and pathway-specific protein analysis, we examined changes in nutrient sensing and unfolded protein response (UPR) pathways known to be influenced in intrauterine growth restriction (IUGR). We also employed targeted metabolomic analysis of amino acids and one-carbon metabolites in maternal blood in association with ambient temperature. Finally, we evaluated the impact of maternal nutritional supplementation (MNS) on HS-associated effects on intrauterine growth. Our results suggest that in the setting of prevalent maternal malnutrition, higher ambient temperature in the first trimester is associated with lower birth length and persistent transcriptomic changes in the placenta. Importantly, our results suggest that improving maternal nutritional status may provide resilience against the effects of HS on fetal growth highlighting the interactions between nutritional and environmental stressors.

## Materials and methods

### Study participants and procedures

This study was an ancillary analysis of a randomized controlled study; the Women First Maternal Preconception Nutrition Trial (WF, clinicaltrials.gov NCT01883193). The WF trial was a multisite randomized trial to determine the efficacy of a comprehensive maternal nutritional supplement (MNS, i.e., micronutrients plus balanced energy supplement) on birth length and other infant anthropometry outcomes at birth (18). The nutritional supplement consisted of a daily 20 g small quantity-lipid-based nutritional supplement (sqLNS) with 22 micronutrients in amounts appropriate for pregnancy and lactation (Table S3). In addition to the multiple micronutrients and polyunsaturated lipids (linoleic 4.9 g and α-linolenic 0.59 g), the composition included dried skimmed milk, soybean and peanut extract, sugar, maltodextrin stabilizers, and emulsifiers (Nutriset, Malauney, France). An additional lipid-based balanced protein/energy supplement (300 kcal/d; 12% calories from protein and no added micronutrients; Nutriset, Maluaney, France) was provided to women with low BMI (≤20) or low gestational weight gain (18). The trial was conducted in rural or semi-rural locations in India, Pakistan, Guatemala, and the Democratic Republic of the Congo and included three arms: Arm 1, women consumed MNS ≥ 3 mo prior to conception until delivery; Arm 2, women consumed the same MNS commencing at 12 wk gestation until delivery; or Arm 3, no MNS was administered, and women followed local pregnancy standard of care. The trial and primary outcomes have been previously described in detail (18, 42). The WF study protocol received IRB approval at the University of Colorado and local ethics committees granted approval at each trial site.

The present analysis focused on a subcohort of mothers from the Pakistan site for whom ultrasound based gestational age was available (*n* = 459). Participants were categorized into three groups according to season of birth: winter (November to February), summer (March to June), and rainy (July to October). The study design is shown in Fig. 1. Maternal height and weight were obtained during an in-person study visit by on-site study personnel who were trained to measure subject anthropometrics using an electronic scale and adult stadiometer. Preconception weight status was defined as underweight (BMI < 18.5 kg/m^2^), normal weight (BMI 18.5 to 24.9 kg/m^2^), overweight (BMI 25 to 29.9 kg/m^2^), and obese (BMI > 30 kg/m^2^). SES indicator score was calculated as a score from 0 to 6, with 6 indicating the highest SES status (42).

### Anthropometric measurements

Trained assessment teams measured women and newborns using standardized equipment and procedures. Maternal height and weight were measured at enrolment. Newborn anthropometry was measured within 48 h after delivery. Newborn weight was measured in triplicate using a Seca 334 electronic scale (seca North America, Chino, CA, USA) and newborn length was measured using a neonatal stadiometer (Ellard Instrumentation Ltd, Monroe, WA, USA). Gestational age was determined by first-trimester ultrasound using crown-rump length. For these analyses, we converted birth length, weight, and head circumference to gestational-age adjusted *Z*-scores (LGAZ, WGAZ, and HCGAZ, respectively) based on INTERGROWTH-21st standards (22).

### Derivation of ambient heat and environmental exposures

Daily relative humidity, mean and maximum air temperature for the entire study duration were acquired from the closest automated surface observation systems using the GSODR package (Fig. S1). GSODR is a set of data mining tools that facilitates finding, transfer, and formatting of meteorological data (National Centers for Environmental Information) (62). We calculated the average daily maximum temperature (avg *T*_max_) for each infant for three 90-day windows representing each trimester of gestation, referred to as avg *T*_max_ for trimesters 1, 2, and 3. We also calculated the number of days in each developmental window when the *T*_max_ was >39°C to reflect “HS days.” Based on the distribution of HS days, we also discretized exposure to “excessive HS” as a categorical variable when the number of HS days was greater than 20 in any given 90-day window. We also derived heat index values based on average daily temperature and relative humidity levels (using the Rothfusz equation implemented in the weathermetrics R package) (23). Similar to average *T*_max_ for each trimester, average heat index for each trimester was calculated and used as the exposure variable. Levels of ambient fine particulate matter <2.5 μm, (PM_2.5_ levels) were derived from the Modern-Era Retrospective Analysis and Research and Application version 2 product (MERRA-2 analysis. MERRA-2 utilizes satellite- and ground-based data for atmospheric chemistry to derive aerosol optical depth of five aerosols including dust, sea salt, sulfate (SO_4_), black carbon, and organic carbon levels that contribute to derivation of PM_2.5_ levels (μg/m^3^) (63). Hourly values throughout the globe at 0.5° × 0.65° (∼55 × 72 km) spatial resolution are available from 1980 onwards. These values were compiled for the Thatta region (24.75 lat, 67.91 lon) and daily average values were calculated for the duration of the study (2013 to 2016, Fig. S1).

### Placental gene expression analysis

Placental dimensions were obtained after delivery by alignment of the longest axis of the placenta (length) on a grid and recording the widest point perpendicular to the longest dimension axis (width). Placental tissue was collected from four representative locations (1 cm^3^ each), washed, and immersed into PBS with protease and phosphatase inhibitors for protein analysis or RNA*later* for gene expression analysis (Thermo Fisher Scientific, Waltham, MA, USA). Samples were processed according to manufacturer’s instructions and stored at −80°C until further analysis. Collection time and storage temperature were recorded until samples were shipped to the University of Colorado, Denver, USA. Total RNA was isolated from villous placenta stored in RNA*later* using a combination of TRI reagent (Molecular Research Center, Cincinnati, OH, USA) and RNeasy-mini columns, including on-column deoxyribonuclease digestion (Qiagen, Germantown, MD, USA) (64, 65). Directional cDNA libraries for mRNA-sequencing were prepared using polyA-mRNA from individual RNA samples using Illumina TruSeq reagents (64, 65). Detailed methods are described in Supplementary Materials. Pooled cDNA libraries were sequenced (150 bp paired reads) using a NovaSeq 6000 instrument (Illumina, San Diego, CA, USA). Data analysis is described in the statistical analyses section.

### Immunoblotting

Total placental tissue lysates (*n* = 12 per group for HS and non-HS controls) were prepared in radio-immunoprecipitation assay (RIPA) buffer supplemented with 1 mM PMSF, protease and phosphatase inhibitor cocktails (Halt inhibitors Detailed procedures are included in supplementary materials. Immunoblotting was carried out using the ProteinSimple Wes electrophoresis system (bio-techne, Minneapolis, MN, USA). Samples were probed with antibodies specific for phospho-EIF2α, total EIF2α, phospho p70-S6K, total p70-S6K, phospho 4EBP-1, total 4EBP-1, phospho-AMPK and total AMPK and vinculin (1:1000 dilution, all antibodies were procured from Cell Signaling Technologies Inc., Danvers, MA, USA). Proteins were visualized using labeled rabbit secondary antibodies and quantitated using the Compass software (ProteinSimple). For each lane, densitometric value for each protein was normalized to the value of vinculin control. Data were expressed as ratio of phosphorylated to total levels of each protein.

### Blood metabolite analysis using dried blood spot cards

Nonfasting blood samples were collected by venipuncture at 34 wk gestation (*n* = 131). Approximately 0.5 mL of whole blood was applied to a Whatman 903 protein saver dried blood spot (DBS) card (GE Healthcare Life Sciences). DBS cards were then stored at −20°C with desiccant packs and humidity indicator cards. A targeted quantitative one-carbon and amino acid analysis panel was performed using LC–MS/MS at the Southeast Center for Integrated Metabolomics (24). The standard panel consisted of 36 amino acids, methylated amino acids, and 1C metabolites. Detailed analytical methods are described in Supplementary Materials. Metabolite concentrations were calculated by comparing these peak area ratios to standard curves prepared using authentic standards.

### Statistical analysis

All statistical analyses were conducted in R statistical software (v4.1). Data are expressed as mean ± SD for descriptive analysis of the cohort. Statistical comparisons of descriptive characteristics based on season of birth were performed using one-way ANOVA or chi-squared testing. Continuous outcome variables including LGAZ, WGAZ, and HCGAZ were adjusted using INTERGROWTH-21st standards (22). Normality of distributions of the outcome variables were confirmed using the Shapiro–Wilk test. Outliers were identified as values outside 1.5 times the interquartile range (1.5× IQR). Exposure variables included season of birth, average maximal daily temperature (*T*_max_) for each trimester, number of days with *T*_max_ > 39°C in each trimester, categorical exposure to excess HS (defined as over 20 days with *T*_max_ > 39°C), and heat index values. Associations between season or temperature exposure variables and outcomes were assessed using multiple linear regression. Models were adjusted for recruitment site cluster (model 1); cluster, maternal age, and parity (model 2); or model 2 covariates plus infant sex, mode of delivery, MNS treatment arm, and maternal weight gain between 12 and 32 wk of pregnancy (model 3). Finally, model 4 included model 3 plus relative humidity and PM_2.5_ as covariates in association analysis between average maximal daily temperature (*T*_max_) for each trimester and birth outcomes. Model parameters (β-coefficient, 95% CI and *P*-values) were derived using the *parameters* package. A nominal *P*-value of *P* < 0.016 was considered statistically significant based on a conservative Bonferroni correction for three outcomes (*P* < 0.05/3 = 0.016). To examine the modification of HS effects in the first trimester on LGAZ, WGAZ, and HCGAZ multiple linear regression was performed including supplementation status as an independent variable along with excess HS and other covariates (cluster, maternal age, parity, infant sex, mode of delivery, and gestational weight gain). Additional models were performed on data stratified by nutrition supplementation arm. Arms 2 and 3 were designated as nonsupplemented groups (as intervention in Arm 2 began after week 12 of pregnancy), while arm 1 was supplemented with comprehensive nutrition prior to conception and the first trimester. In addition, to compare groups with similar sample sizes, linear regression of birth outcomes was performed with excess HS and all nutritional intervention arms separately along with above mentioned covariates. For visual representation in violin plots excessive HS vs. control groups were compared via Wilcoxon test using the ggpubr package.

#### RNA-seq data analysis

Following sequencing and demultiplexing, reads were trimmed for adapters and filtered based on quality score using Fastp as previously described (64, 65). Raw and processed data are available at the Gene Expression Omnibus repository as GSE220877. High quality reads were aligned to the human genome (hg19) using STAR with default settings. Resulting read alignments for each sample were imported in Seqmonk for transcript level quantification as counts mapping to annotated genes. Correlations between PCs and *T*1–*T*_max_ was done using the corrplot package. Volcano plots showing all DEGs, dotcharts for top DEGs and PCA plots were created in R. Absolute counts mapping to genes were imported into R and analysis of differential expression between groups was done using the limma-voom pipeline that includes steps for preprocessing, linear modeling, and differential expression analysis (66). Genes with expression below 0.5 cpm (counts per million mapped reads) were excluded from further analysis. DEGs between HS and non-HS controls in the first trimester were identified using functions in the limma package (66) with an FDR *P*-value < 0.05 and a minimum fold change ± 1.4 fold. Models were adjusted for batch of library preparation, sex of the placenta, and MNS treatment. Sex-stratified analysis was carried out separately to examine effects of excess HS in male and female placenta separately. Venn lists were created using the JVenn plugin. Interpretive analysis of DEGs for enrichment of gene ontology (GO) terms, pathway, and upstream regulator analysis was done using Enrichr and IPA software (67). Barplots representing top enriched GO terms were made using the barplot function using results from Enrichr analysis. Normalized gene expression values for specific gene sets (ribosomal RPL/RPS genes and those involved in UPR) were extracted from normalized data and plotted using the ggpubr package.

#### Placental protein expression analysis

Levels of phosphorylated p70-S6K, EIF2a, 4EB-P1, and AMPK were expressed as relative ratios against respective total proteins. Log-transformed data was employed to compare groups via Student’s *t*-test.

#### Metabolomic data analysis

Metabolite concentrations were derived from absolute standards and expressed as μg/ml in blood. Metabolites with missing concentration measurements for >10% of samples were removed from further analysis. Metabolite concentrations were converted to log-transformed normalized values (*Z*-scores). Statistical outliers were defined as 1.5× the IQR below or above the first or third quartiles, respectively. Seven metabolites (carnitine, deoxycarnitine, glutamate, glycine, homocysteine, leucine, and methionine sulfoxide) had missingness > 10% and were removed from further analysis. PCs analysis for metabolites was done using the FactoMineR and factoextra packages. Associations between average daily maximal temperature (*T*_max_) in the first trimester and metabolite concentrations at 34 wk of pregnancy were assessed via linear regression. Models were adjusted for recruitment site cluster and nutrient supplementation Arm. False-discovery rate adjusted *P*-values were calculated to account for multiple hypothesis testing. Metabolites that were significantly associated with ambient temperature (FDR adjusted *P*-value < 0.1) were used for metabolite overrepresentation analysis using MetaboAnalyst (68). Scatter plots of select metabolites and ambient temperature were generated using the ggpubr package.

## Results

### Cohort characteristics

This analysis included data from 459 women and their neonates studied between December 2013 and September 2016. The average maternal age at enrolment was 23.6 ± 4.1 y and BMI was 19.7 ± 2.9 kg/m^2^. Based on the season in which delivery of the neonate occurred, sample sizes were 149 mother-infant pairs in winter (November to February), 143 in summer (March to June), and 167 in the rainy season (July to October) (Table S1). Maternal age, weight, height, BMI, mode of delivery, hemoglobin concentrations, socio-economic status (SES), food insecurity, study arm, compliance of supplement use, and infant sex distribution did not significantly differ by season of birth. Only parity was significantly different between seasons, with participants giving birth in November to February (season 1) showing lowest percentage of nulliparous mothers (21.2%) and season 3 showing highest percentage (39.1%). Distribution of participants by treatment arms and composition of the nutritional supplement are presented in Tables S2 and S3, respectively.

### Season of birth impacts birth length and head circumference

**Fig. 2.**
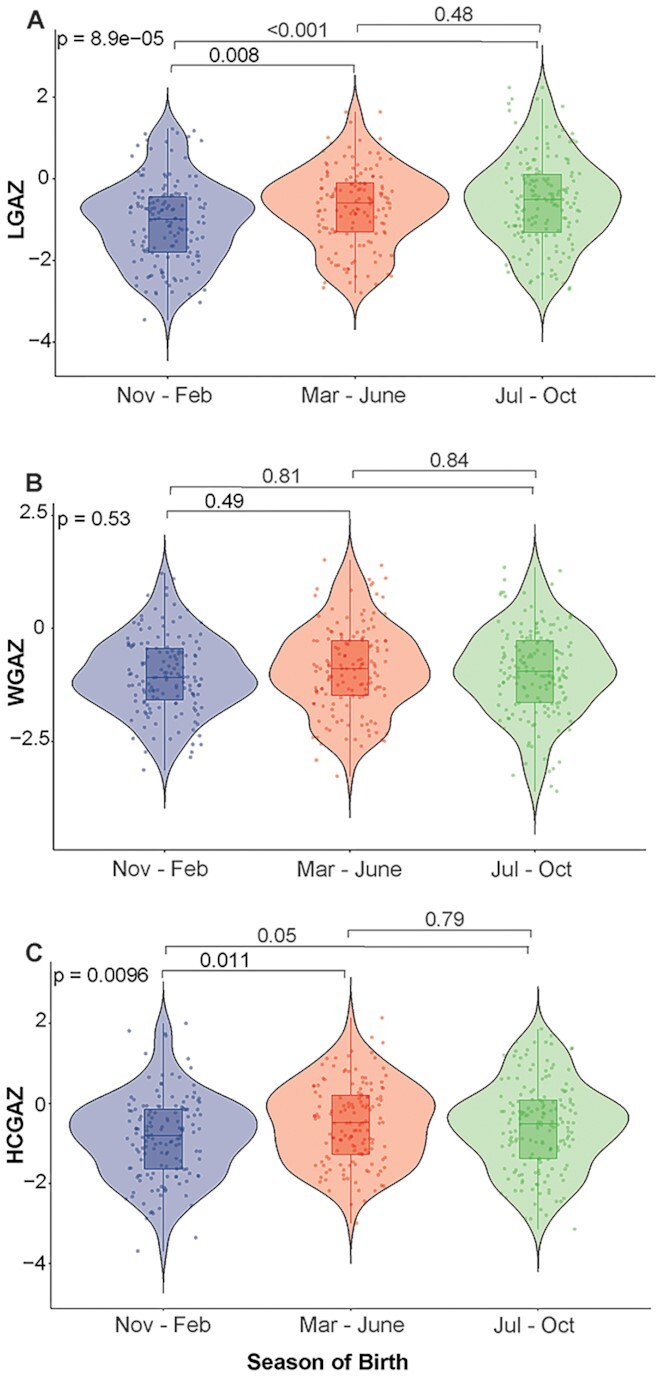
Seasonal impacts on neonatal birth outcomes. Violin plots showing neonatal (A) LGAZ, (B) WGAZ, and (C) HCGAZ measured within 48 h of birth. Anthropometric measures were adjusted using the INTERGROWTH 21st standards. Infants were categorized into seasons based on month of birth. Nov to Feb is winter (*n* = 149), Mar to June is summer (*n* = 143), and July to October is rainy season (*n* = 163), respectively. LGAZ, length-for-age *Z*-score; WGAZ, weight-for-age *Z*-score; and HCGAZ, head circumference-for-age *Z*-score. Groups were compared via one-way ANOVA followed by post-hoc comparisons using Tukey HSD. Results of covariate-adjusted linear regression models are presented in tables.

Neonatal outcomes of birth weight, length, and head circumference were converted to gestational-age-appropriate *Z*-scores using the INTERGROWTH-21st standards (22) (referred to as WGAZ, LGAZ, and HCGAZ). Linear regression of these outcomes against season of birth and adjusting for covariates showed strong associations for LGAZ and HCGAZ (Table 1). Being born in the winter was associated with smaller birth length compared to summer (β = 0.35, 95% CI = 0.11 to 0.59, *P* = 0.004) and rainy season (β = 0.47, 95% CI = 0.23 to 0.70, *P* < 0.001). Likewise, infants born in the winter showed lower HCGAZ compared to summer (β = 0.35, 95% CI = 0.10 to 0.59, *P* = 0.005), and rainy season (β = 0.27, 95% CI = 0.04 to 0.51, *P* = 0.02) (Table 1, Fig. 2). Neonatal WGAZ was not significantly associated with season of birth.

**Table 1.** Associations between season of birth and neonatal anthropometric outcomes.

Outcome	Exposure	β	SE	CI (low)	CI (high)	*P*-value
	*Season of birth*					
LGAZ	Summer	0.3542	0.1236	0.1112	0.5971	**0.0043**
LGAZ	Rainy	0.4715	0.1191	0.2373	0.7056	**0.0000**
WGAZ	Summer	0.1802	0.1091	−0.0343	0.3948	0.0994
WGAZ	Rainy	0.1232	0.1053	−0.0837	0.3303	0.2426
HCGAZ	Summer	0.3502	0.1259	0.1026	0.5978	**0.0056**
HCGAZ	Rainy	0.2799	0.1212	0.0415	0.5183	**0.0214**

Multiple linear regression models for each birth outcome were assessed independently. Models were adjusted for maternal age, parity, and recruitment cluster. Births in winter were the reference class.

Abbreviations: LGAZ, length-for-age *Z*-score; WGAZ, weight-for-age *Z*-score; and HCGAZ, head circumference-for-age *Z*-score.

### HS detrimentally impacts intrauterine growth

**Fig. 3.**
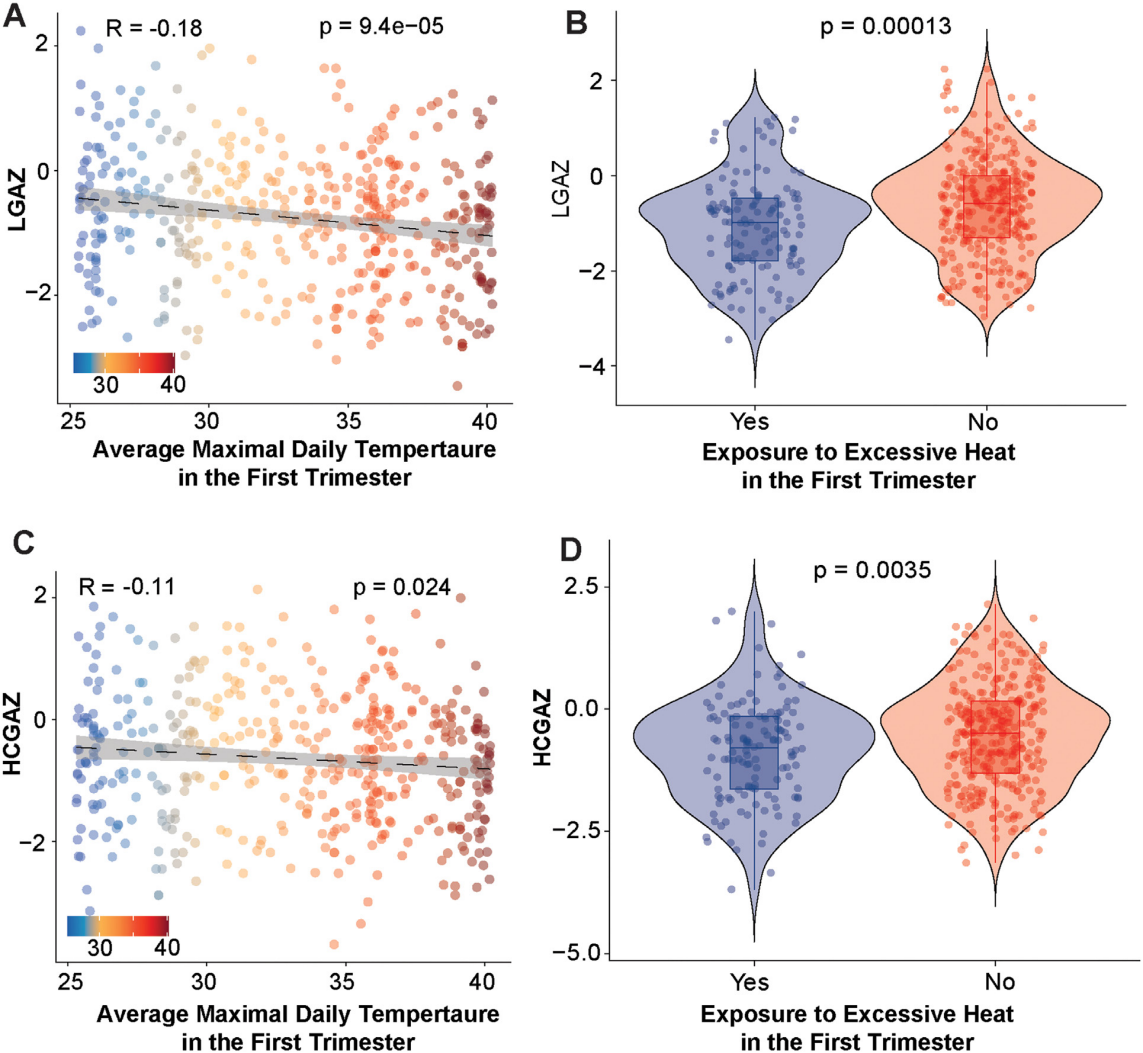
Association between heat exposure in the first trimester and birth anthropometry. Scatter plots showing association between average daily maximal temperature (*T*_max_) in the first trimester and (A) LGAZ and (C) HCGAZ at birth. *T*_max_ values for each participant were calculated using data from local weather stations. Violin plots (B) LGAZ and (D) HCGAZ in neonates exposed to excess HS during the first trimester. Excessive HS was defined as >20 days with *T*_max_ over 39°C in the first trimester. LGAZ, length-for-age *Z*-score; WGAZ, weight-for-age *Z*-score; and HCGAZ, head circumference-for-age *Z*-score. *P*-values were derived using Pearsons correlation or Wilcoxon test. Results of covariate-adjusted linear regression models are presented in tables. Samples sizes were *n* = 130 and 321 in excess HS and non-HS groups, respectively.

We evaluated associations between recorded ambient temperature in defined developmental windows and birth outcomes. Profiles of maximal daily temperature, relative humidity, heat index, and PM_2.5_ levels over the year are shown in Fig. S1. Infants born in the winter had an average of 35.4 days with *T*_max_ > 39°C in the first trimester (90-day window), distinctly more (*P* < 2.2e^−16^) than infants born in the other seasons reflecting strong heat exposure in the first trimester of development (Fig. S2). Linear regression models examining associations between heat exposure and outcome variables (LGAZ, WGAZ, and HCGAZ) were adjusted for important covariates that included geographical cluster, maternal age, parity, infant sex, mode of delivery, maternal weight gain during pregnancy, nutritional intervention, relative humidity, and levels of fine particulate matter (PM_2.5_). Heat exposure variables included three metrics: (1) average maximal daily temperature (*T*_max_) in each trimester; (2) the number of days when *T*_max_ > 39°C; and (3) exposure to excessive HS in a trimester. Excessive HS was categorically defined as >20 days with *T*_max_ > 39°C in a given trimester. Linear regression models showed a strong negative association between *T*_max_ in the first trimester and LGAZ, which remained significant in the fully adjusted model adjusted for cluster, maternal age, parity, infant sex, mode of delivery, gestational weight gain, and nutritional intervention (model 3, β = −0.039, 95% CI = −0.06, −0.01, *P* = 0.0001, Table S4, Fig. 3A). Similar negative associations were also observed with HCGAZ and first trimester *T*_max_ (β = −0.029, 95% CI = −0.05, −0.008, *P* = 0.005, Table S4, Fig. 3C). For each 5°C increase in the *T*_max_ in the first trimester, LGAZ decreased by 0.19 *Z*-scores and HCGAZ decreased by 0.14 *Z*-scores. Ambient temperature in the second trimester was not associated with LGAZ, WGAZ, or HCGAZ. However, *T*_max_ in the third trimester was positively associated with LGAZ, albeit with smaller magnitude (β = 0.03, 95% CI = 0.009, 0.05, *P* = 0.005, Table S4). We also examined if relative humidity and PM_2.5_ levels modified the associations between heat and birth outcomes. Inclusion of average relative humidity and PM_2.5_ concentrations did not alter significant associations between *T*_max_ and LGAZ (model 4, β = −0.039, 95% CI = −0.06, −0.017, *P* = 0.0004, Table S4). To assess the cumulative impacts of heat and relative humidity, we calculated heat index values using the Rothfusz equation (23). Heat index in the first and second trimesters was negatively associated with LGAZ and HCGAZ (Table 2). Analysis of severe heat exposure variables (days > 39°C and excessive HS) broadly confirmed associations with LGAZ. Both the number of days > 39°C and exposure to excessive HS in the first trimester were associated with lower LGAZ (Tables S5 and S6, Fig. 3B). Likewise, number of days > 39°C and exposure to excessive HS in the first trimester were also associated with lower HCGAZ (Tables S5 and S6, Fig. 3D). Overall, HS during the first trimester was negatively associated with birth outcomes.

**Table 2.** Associations between exposure to heat index in pregnancy and infant anthropometric outcomes at birth.

Outcome	*Trimester (Heat index)*	β (95% CI)^a^	*P*-value
LGAZ	T1—HI	−0.024 (−0.038, −0.009)	**0.001**
	T2—HI	−0.019 (−0.033, −0.005)	**0.008**
	T3—HI	0.018 (0.002, 0.034)	0.021
WGAZ	T1—HI	−0.007 (−0.02, 0.005)	0.266
	T2—HI	−0.009 (−0.021, 0.003)	0.158
	T3—HI	0.005 (−0.007, 0.019)	0.404
HCGAZ	T1—HI	−0.020 (−0.035, −0.005)	**0.007**
	T2—HI	−0.017 (−0.032, −0.002)	**0.018**
	T3—HI	0.021 (0.005, 0.037)	**0.007**

Abbreviations: LGAZ, length-for-age *Z*-score; WGAZ, weight-for-age *Z*-score; and HCGAZ, head circumference-for-age *Z*-score.

Heat index (HI) values were calculated using Rothfusz equation.

a Models were adjusted for cluster of recruitment, maternal age, parity, infant sex, mode of delivery, and maternal weight gain between 12 and 32 wk of pregnancy and PM_2.5_ concentrations.

### Preconception nutritional supplementation modifies effects of HS

**Fig. 4.**
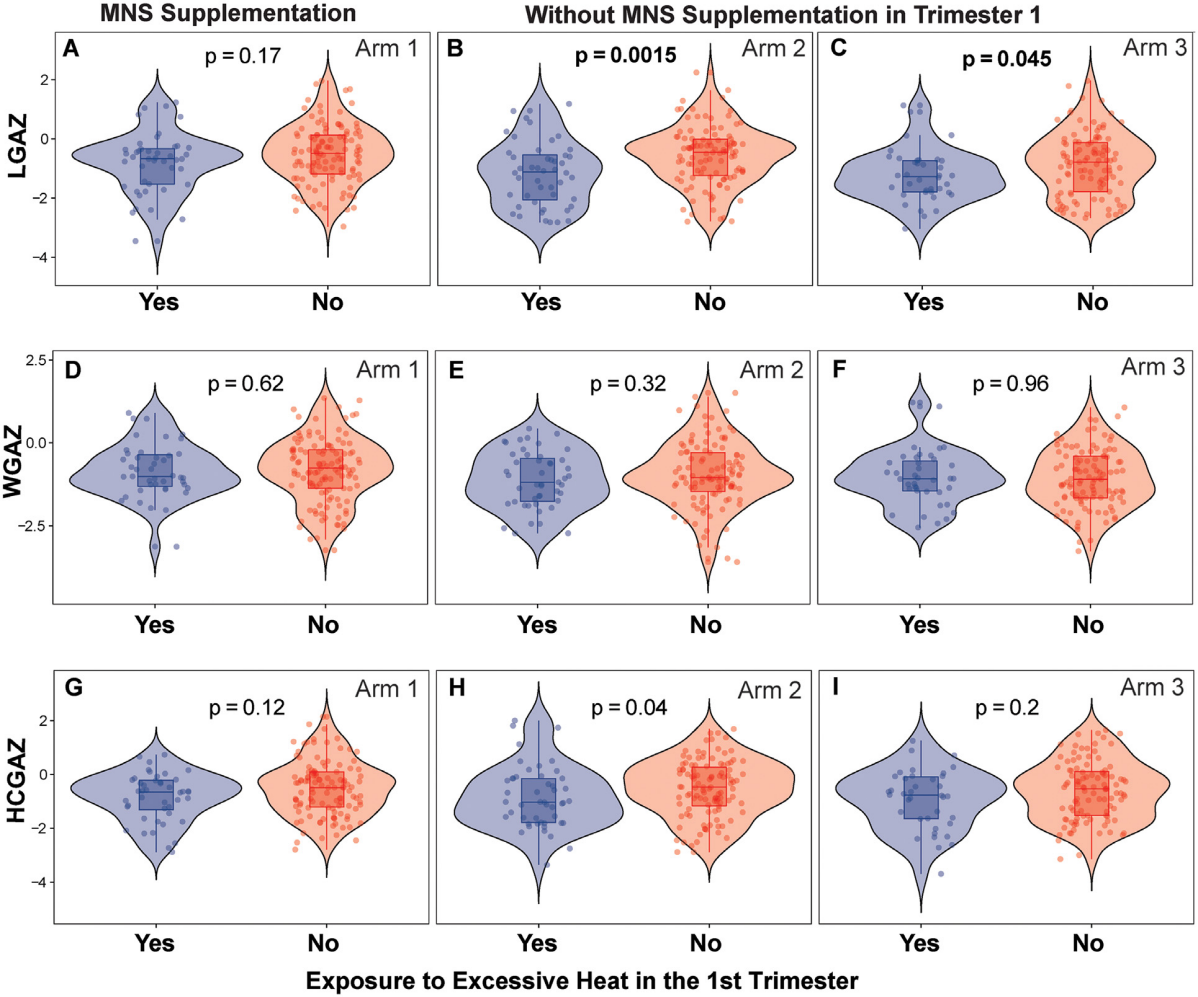
Association between excessive HS and birth anthropometry stratified by MNS. (A to C) LGAZ, (D to F) WGAZ, and (G to I) HCGAZ at birth of infants born to mothers who were either unsupplemented (Arms 2 and 3) or supplemented (Arm 1) with MNS prior to conception. Excessive HS was defined as >20 days with *T*_max_ over 39°C in the first trimester. Samples sizes in HS and non-HS groups were *n* = 44 and 114 in Arm 1; *n* = 48 and 106 in Arm 2; and *n* = 38 and 101 in Arm 3, respectively. *P*-values were derived using Wilcoxon test. Results of covariate-adjusted linear regression models are presented in Table 3.

Poor nutritional status is likely to exacerbate HS-associated growth retardation. To examine changes in heat-associated effects with nutritional supplementation, we examined LGAZ, WGAZ, and HCGAZ at birth by supplementation group. Both Arms 2 and 3 received no MNS during the first trimester (as the supplementation in arm 2 began at the end of the first trimester) and hence were considered unsupplemented relative to heat exposure in the first trimester. We focused on excess HS as the exposure variable (categorically defined as >20 days of *T*_max_ > 39°C). Multiple linear regression of excess HS in the first trimester against birth outcomes (including covariates cluster, maternal age, parity, infant sex, mode of delivery, and gestational weight gain) showed a strong effect of heat (β = −0.494; *P* = 0.0002) but not a significant interaction with MNS (β = 0.244; *P* = 0.271). However, in linear regression models stratified by supplementation status, significant effects of HS on LGAZ and HCGAZ were observed in unsupplemented women but not in those provided MNS prior to conception (Table S7, Fig. S3). To avoid unbalanced sample sizes between supplemented and unsupplemented groups, we also considered all intervention arms separately. For LGAZ as the outcome, interaction between excess HS and interventions Arms 2 and 3 did not reach significance (*P* = 0.23 and 0.68, respectively). In models stratified by treatment Arms however, significant negative associations between LGAZ and heat were observed in Arms 2 and 3, but not in Arm 1 (Table 3). Consistent with these findings, Wilcoxon tests show lower LGAZ in neonates from women exposed to HS in Arms 2 and 3 (no MNS supplementation), but nonsignificant effects in HS mothers receiving MNS (violin plots, Fig. 4A to C). For head circumference, effects of HS were comparable across intervention arms (Table 3, Fig. 4G to I), suggesting limited effect of preconception intervention on HCGAZ when considering similar sample sizes. Overall, the findings suggest that preconception nutritional supplementation may mitigate the effects of heat on birth length.

**Table 3.** Associations between excess HS and infant anthropometric outcomes stratified by MNS treatment Arm.

Outcome	Arm 1 (MNS supplementation) *N* = 158		Arm 2 (No MNS supplementation) *N* = 154		Arm 3 (No MNS supplementation) *N* = 139	
	β (95% CI)	*P*-value	β (95% CI)	*P*-value	β (95% CI)	*P*-value
LGAZ	−0.277 (−0.637, 0.081)	0.12	−0.667 (−1.040, −0.294)	**0.0005**	−0.505 (−0.934, −0.082)	**0.01**
WGAZ	−0.026 (−0.355, 0.303)	0.87	−0.378 (−0.739, 0.016)	0.04	−0.095 (−0.443, 0.253)	0.58
HCGAZ	−0.383 (−0.742, −0.024)	0.03	−0.391 (−0.789, 0.006)	0.05	−0.398 (−0.845, −0.048)	0.07

Models were adjusted for cluster, maternal age, parity, infant sex, mode of delivery, and maternal weight gain between 12 and 32 wk of pregnancy. Excessive HS was defined as >20 days with *T*_max_ over 39°C in the first trimester. Abbreviations: LGAZ, length-for-age *Z*-score; WGAZ, weight-for-age *Z*-score; and HCGAZ, head circumference-for-age *Z*-score. Samples sizes in HS and non-HS groups were *n* = 44 and 114 in Arm 1; *n* = 48 and 106 in Arm 2; and *n* = 38 and 101 in Arm 3, respectively.

### Early pregnancy heat exposure impacts the placental gene expression

**Fig. 5.**
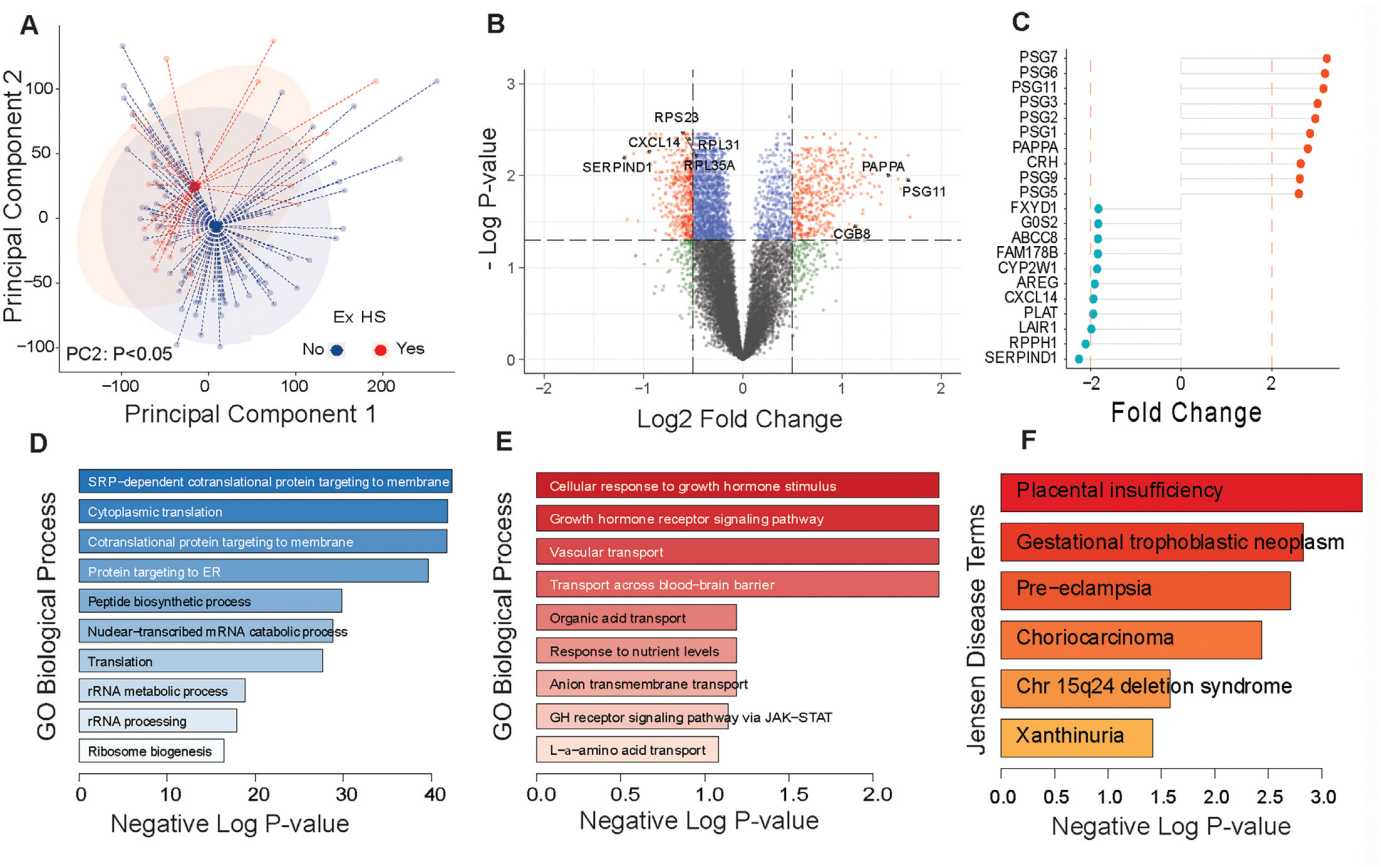
Placental gene expression changes associated with excessive HS. Global transcriptomic analysis of term villous placentas was conducted using mRNA-sequencing (*n* = 126). Data analysis to identify DEGs was done based on excessive HS in the first trimester (*n* = 29 HS; *n* = 97 non-HS controls). Excessive HS was defined as >20 days with *T*_max_ over 39°C in the first trimester. (A) PCs analysis of all expressed genes shows significant effect based on HS (PC2, Student’s *t*-test *P* < 0.05). (B) Volcano plot showing all DEGs, and (C) dot charts of showing top DEGs affected by HS. GO Biological process enrichment among (D) downregulated and (E) upregulated DEGs with HS using Enrichr showing highly significant enrichment of protein translation genes. (F) Enrichment of Jensen disease terms using text mining via Enrichr showing enrichment of placental insufficiency, pre-eclampsia, and placental disease among upregulated genes.

**Fig. 6.**
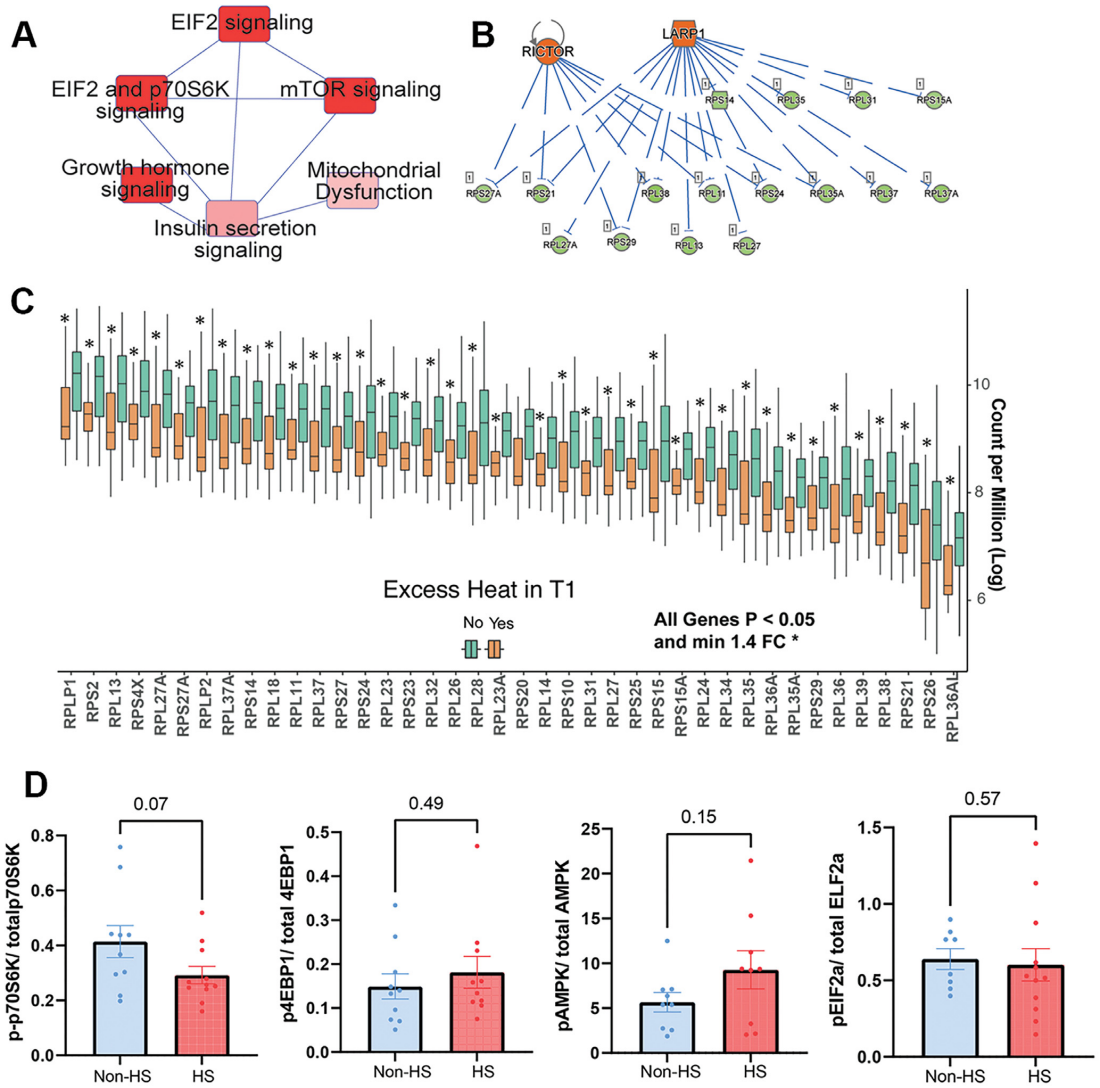
Placental gene network and protein expression changes associated with excessive HS. Global transcriptomic analysis of term villous placentas was conducted using mRNA-sequencing (*n* = 126). Data analysis to identify DEGs was done based on excessive HS in the first trimester (*n* = 29 HS; *n* = 97 non-HS controls). Excessive HS was defined as >20 days with *T*_max_ over 39°C in the first trimester. (A) IPA based analysis of DEGs showing enrichment of mTOR and downstream targets. (B) Upstream regulator analysis showing predicted regulation of RPS and RPL genes via mTOR and LARP1. (C) mRNA expression of critical RPL and RPS family genes that play critical roles in protein translation showing coordinated downregulation in HS placenta. (D) Protein expression of pEIF2a, p-70S6K, p-4EBP1, and pAMPK, relative to total levels of respective protein in placental whole cell lysates (*n* = 12/group). *P*-values were derived using Student’s *t*-test. Complete list of DEGs included in Supplementary Materials.

We utilized mRNA-sequencing to examine placental gene expression changes associated with excessive HS in the first trimester. A subsample from the full cohort for which placenta were successfully collected were included in this analysis. Characteristics of this subsample were similar to the full analytical cohort except for a significant difference in participants age (22.7 ± 4.1 vs. 23.6 ± 4.1 y, mean ± SD for placental subset and full cohort, respectively, Table S8). Samples were grouped into those exposed to excess HS or nonexposed controls (*n* = 29 excessive HS; *n* = 97 non-HS controls). Classification into the HS vs. non-HS groups was determined by the number of HS days (days with *T*_max_ > 39°C) in the first trimester. A participant was assigned to the excess HS group when exposed to >20 HS days within the first trimester. Collectively, the RNA-seq analysis included 4.53 billion paired-reads. Correlation analysis of all principal components (PCs) with first trimester *T*_max_ showed significant correlation with PC2 (*P* = 0.0007, Table S9). PC analysis of all expressed genes showed a significant effect of early HS along PC2 (Student’s *t*-test *P* < 0.05, Fig. 5A). Identification of differentially expressed genes (DEGs) (adjusted for placental sex and supplementation arm) revealed that early HS was associated with altered expression of 995 genes (FDR *P* < 0.05; ± 1.4-fold change, Table S9 and Fig. 5B). Top DEGs altered with HS are plotted in Fig. 5C. We utilized Enrichr and Ingenuity Pathway Analysis (IPA) to functionally interpret DEGs. GO biological processes enriched among genes decreased with HS included those involved in protein targeting to endoplasmic reticulum (ER), protein biosynthesis, SRP-dependent co-translation, and cytoplasmic translation were highly significantly enriched (adj *P*-value = 1.9e^−35^) (Fig. 5D, Table S10). Among genes increased with HS, GO terms related to cellular response to growth, vascular transport, and nutrient transport were increased (Fig. 5E, Table S11). Text mining of the DISEASES resource (enrichment of Jensen Disease terms) among genes upregulated with HS showed enrichment of placental insufficiency, pre-eclampsia, and trophoblastic disease (Fig. 5F, Table S12). KEGG pathway analysis also showed that ribosome pathways were by far the most affected (adj-*P* = 9.4e^−31^). Analysis via IPA indicated that EIF2A, p70S6K, and mTOR signaling were strongly associated with HS (Fig. 6A). Upstream regulator analysis in IPA showed alterations in RPL/RPS genes were predicted to be strongly associated with changes in mTOR complex component Rictor and LARP-1, a critical regulator of protein translation (Fig. 6B). This included a coordinated reduction in the expression of 43 RPL/RPS family transcripts (Fig. 6C), which are ribosomal proteins with key roles in ribosome biogenesis and protein translation. mRNA expression of key regulators of the UPR (ER stress) including XBP1, HSPA5 (BiP), and ERN1 (Ire1) were significantly upregulated (*P* < 0.01) following HS (Fig. S4). Since gene expression data suggested decreased mTOR/TORC1 signaling, we examined protein and phosphorylation status of two key downstream targets of TORC1 signaling, p70-S6K and 4EBP-1, along with AMPK and EIF2α, which are sensitive to ATP levels and the PERK axis of ER stress, in a subset of placenta samples (*n* = 12/group). Phosphorylation of 4EBP-1, EIF2α, and AMPK was not altered significantly, but phosphorylation of p70S6K was lower in HS placenta (*P* = 0.07) (Fig. 6D). While the primary analysis was adjusted for placental sex, we also carried out sex-stratified analysis of placental gene expression changes in male and female sexes separately. Sample sizes in the non-HS controls and HS groups were 56 and 17 in females, and 41 and 12 in males, respectively. Despite these relatively small sample sizes, we observed robust sex differences in the effect of HS compared to non-HS groups in males vs. females. PCA showed significant separation in male placenta (PC2, *t*-test *P* < 0.05) but not in females. We identified 3075 DEGs with HS in males vs. 565 in females (unadjusted *P* < 0.05 and ± 1.4-fold change, Fig. S5, Tables S13 and S14). Using FDR adjusted *P*-values (adjusted-*P* < 0.05 and ± 1.4-fold change), there were 1533 DEGs in males and no DEGs in females meeting these criteria. We also examined overlap between DEGs in male and female placenta (Table S15, Fig. S5) and identified 283 commonly regulated transcripts. GO biological process enrichment for these 283 common genes also showed strong enrichment of SRP-dependent co-translation, protein targeting to ER and translation as top terms. Complete lists of DEGs and GO analysis can be found in Table S15. Overall, our findings indicate a consistent effect of first trimester heat exposure on pathways regulating protein translation, ribosomal biogenesis, and ER stress.

Samples for placental transcriptomic analysis included all intervention arms. Hence, all DEGs were adjusted for treatment arm. We also identified independent effects of MNS treatment on placental gene expression. Global gene expression profiles as assessed by PCA did not show separation (Fig. S6A and S6B). Comparisons of effects between Arms 1 vs. 3 (effect of preconception plus gestational MNS vs. unsupplemented controls) and Arms 2 vs. 3 (effect of gestational MNS vs. unsupplemented controls) revealed 125 genes and 30 genes, respectively (unadjusted-*P*-value < 0.05, and ± 1.4-fold change) suggesting very modest changes (Fig. S6C, Tables S16 and S17). Only 10 genes were common among both effects (Table S18). Top DEGs in the preconception MNS treatment included ATP12A, CYP1A1, and TFRC. These genes were enriched for immune response pathways including LDL remodeling, macrophage differentiation, and leukotriene metabolism (Fig. S6 and Table S18).

### Early pregnancy temperature and metabolites in maternal blood

**Fig. 7.**
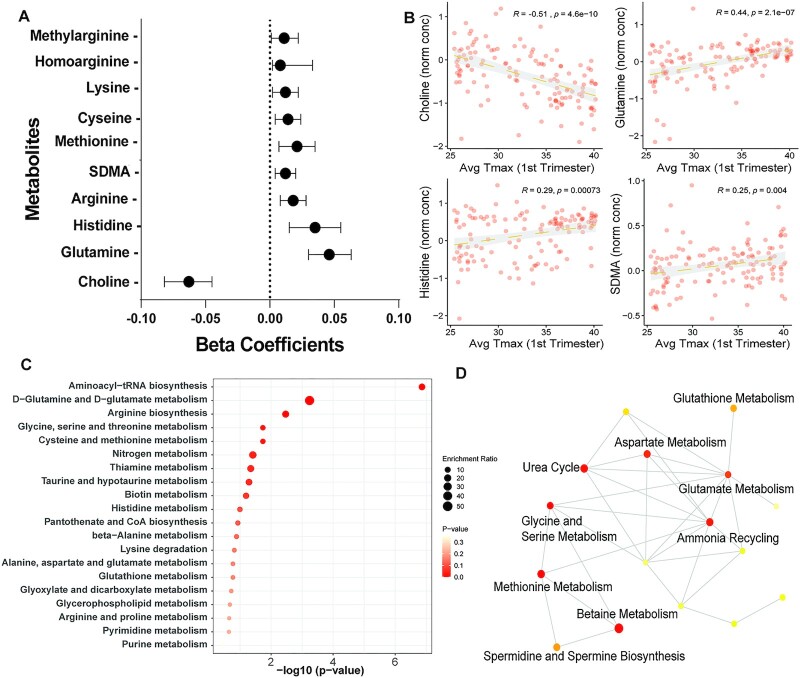
Associations between ambient temperature and maternal blood metabolites. Targeted metabolomics was carried out using LC–MS/MS in blood at 34 wk of pregnancy (*n* = 131). Multiple linear regression was employed to assess the relationship between metabolite levels and ambient temperature in the first trimester. (A) Beta-coefficients and 95% CI for metabolites showing significant associations (FDR adjusted *P*-value < 0.05). (B) Scatter plots showing relationships between metabolite levels of choline, glutamine, histidine, and symmetrical dimethylarginine. R2 and *P*-values derived using Pearson’s correlation. (C and D) Metabolite enrichment analysis and metabolite networks enriched among differentially associated metabolites. Ten metabolites with FDR adjusted *P*-values < 0.1 were utilized for metabolite enrichment analysis.

We examined quantitative associations between avg *T*_max_ in the first trimester and 27 targeted metabolites. Metabolites were measured in maternal blood samples collected at 34 wk of pregnancy. A previous analysis from this cohort showed that while metabolites levels were broadly influenced by pregnancy, very few significant changes occurred with MNS treatment (24). PC analysis of normalized metabolite values did not show broad separation between HS and non-HS groups (Fig. S7). Linear regression models (adjusting for MNS treatment) showed negative associations between ambient temperature and choline levels (Fig. 7A and B; adjusted-*P* = 4.6e^−10^) and positive associations with glutamine, histidine, arginine, methionine, and symmetrical-dimethylarginine levels (Fig. 7A). Complete results of linear regression are included in Table S19. Ten metabolites with FDR *P*-values < 0.1 were included for enrichment analysis using MetaboAnalyst. These showed overrepresentation of aminoacyl t-RNA biosynthesis critical in protein translation, glutamine and glutamate metabolism and arginine biosynthesis (Fig. 7C and D). These results indicate that systemic metabolites change in association with heat exposure may contribute to poor fetal growth.

## Discussion

Climate change represents one of the most pressing threats to child health globally (25). Given the close interconnectedness of climate and nutrition, climate shocks have the potential to slow or even reverse recent gains in improving childhood nutrition and stunting (2, 26, 27). Approximately 820 million children (over one-third of children globally) are currently highly exposed to heatwaves (25). Likewise, the risks for poor pregnancy outcomes associated with ambient heat are of imminent importance, as the increasing frequency and intensity of extreme heat exposures continues unabated (28). Despite wide recognition that ambient heat exposure will continue to impact health of mothers and neonates, relatively few studies have examined this issue in the context of resource-limited settings. In this study, we leveraged maternal and infant anthropometry data from an area acutely affected by both HS and chronic malnutrition. The findings bring into sharp focus the shared impacts of nutritional and environmental stressors on fetal development.

Seasonal changes in pregnancy and birth outcomes are well documented in many populations (29–31). In the closely studied setting of rural Gambia, distinct differences in birth weight, birth length, and subsequent growth, epigenetic alterations and morbidity have been extensively described and associated with the season of birth (32–34). In this setting, lower food availability, greater maternal physical exertion due to seasonal farm work, and exposure to infectious diseases during the hungry season contribute to lower gestation weight gain and nutritional stress-associated growth restriction (31, 33). A recent study from this setting conducted detailed assessments of ambient HS and physiologically derived measures of maternal and fetal strain. The findings documented frequent exposure to extreme HS and greater odds of fetal strain with increasing heat exposure (15). Interestingly, detailed analysis of first trimester diet composition in the Women First study showed high prevalence (>80%) of inadequate dietary diversity and intake of vitamin B12, choline, and folate. However, the analyses did not find seasonal differences in dietary diversity and energy intake (35). Furthermore, associations of ambient temperature with birth length were significant even after adjusting for maternal gestational weight gain (a proxy for energy intake in pregnancy) and did not show seasonal differences in birth weight. Thus, in this context, while further detailed analyses of seasonal environmental, nutritional, and occupational factors are warranted, high-ambient temperature itself was associated with lower birth length and head circumference.

A salient feature of our study is the focus on ambient temperature over critical windows of developmental exposure. The predominant focus of public health interventions in many countries is mitigating risk during extreme heat waves. While this is certainly well-justified to prevent deaths and other serious life-threatening outcomes in high-risk populations, an increasing consensus of studies also points toward detrimental pregnancy and birth outcomes due to higher temperatures, even in the absence of extreme heat events (4, 6). These effects, albeit of small size, may indeed have population level impacts related to greater ambient temperature. A study employing data from the Massachusetts Birth Registry including ∼420,000 birth records showed that ambient air temperature in several windows before birth was negatively associated with birth weight and significant, albeit small, increased odds of preterm birth (36). Likewise, a recent analysis of all live births in Massachusetts showed a negative association between temperatures and term birth weight (37). Conspicuously missing from the literature are measures of birth length, which represents both a key measure of intrauterine linear growth and prognostic measure of stunting risk (38). Our findings suggest that greater ambient temperature in the first trimester negatively impacts birth length, consistent with this developmental window being critical for linear growth. These findings are consistent with a recent report from Ethiopia showing that greater temperatures in the first trimester were positively associated with severe stunting (39). Findings from the MINIMat Study in Bangladesh also showed lower birth lengths of infants born in the winter (November to January) and a negative association with higher temperatures in early gestation (8 wk) (40). Overall, in addition to impacts on birth weight and gestational length, greater temperatures in early pregnancy may contribute to lower linear growth and risk of stunting.

The recognition that nutritional status acts as a modifier of susceptibility to HS is of urgent importance. A large proportion of the world’s population in heat-vulnerable regions also have endemic issues with poor nutrition. This is a particularly pressing issue in the South Asian subcontinent where women of child-bearing age and children have systemic deficiencies in multiple micronutrients and face the double-burden of rising ambient temperatures. In the present context, specific measures of individual-level nutritional status were also measured in the mothers. Both underweight (BMI < 18.5; ∼35% at preconception baseline with mean BMI 19.7) and anemia (72% at baseline) were common in women (41, 42). During pregnancy, women also had high incidence of hypozincemia (∼65 and 74% showed at 12 and 34 wk gestation) and low urine iodine/creatinine ratios (40% in the first trimester) (43). Many studies indicate a collision course of nutritional and climate-related effects on human health. Influence of climate variability such as alterations in precipitation patterns, heat waves, and agricultural pests have macro-level impacts on agricultural and food systems with multiplicative health impacts (44). These effects are magnified in subsistence communities and have been projected to impact nutritional well-being of populations globally. Our findings indicate interactions between effects of ambient heat and nutritional status of mothers prior to conception. Thus, improving nutritional status of mothers may provide resilience against heat-associated effects through biological mechanisms that still need to be understood. In addition to other heat mitigation efforts, nutritional improvement of susceptible populations may be deployed as another mode of developing climate-resilient communities and mitigate risk to pregnant mothers and their children.

Early pregnancy represents a sensitive window during which nutritional and environmental influences have profound effects on fetal development. Evidence primarily from animal models suggest that poor nutrition and HS may converge on similar physiological processes that contribute to diminished fetal growth (viz. placental development) (13, 45). Early pregnancy is also a critical period for establishment of the placenta that is requisite for appropriate nutrient transport in later pregnancy and plays critical roles in long-term developmental programming of offspring health. In sheep, exposure to high ambient temperature leads to placental insufficiency and IUGR (13, 46). Growth restricted offspring also show lower muscle mass, impaired glucose metabolism, and defects in pancreatic development (47–50). Studies in lactating cows, which are prone to HS due to their relatively small surface area: volume ratio, show increased immune cell infiltration, and impaired intestinal barrier (51). Likewise, HS humans and rats have elevated plasma concentrations of proinflammatory cytokines (52, 53).

The molecular mechanisms linking high ambient temperature and diminished growth in humans is much less understood. Results from a prospective birth cohort in Guangzhou, China showed lower placental weight and volume with greater exposure to ambient temperature (17). The present analyses of placental gene expression provide a unique view of transcriptional pathways associated with HS. Notably, a predominant pathway identified here relates to protein synthesis and translation. HS impacts proteostasis, coordinated by pathways linking ER sensing and translation. The ER is essential for protein folding and secretion. The UPR balances new protein synthesis and the folding capacity in the ER. UPR is mediated by three signaling pathways controlled by PERK, IRE1α/XBP-1, and ATF6. While these pathways induce adaptive responses such as ER expansion and folding, unrestrained ER stress leads to inflammation and apoptosis. HS activates UPR, which inhibits heat shock responses via translational inhibition by eIF2α phosphorylation and XBP1 splicing (54). Activation of ER stress antagonizes mTORC1 signaling and in the placenta ER stress is associated with IUGR (55). The mTORC1 complex (one arm of the mTOR pathway) responds to nutrients and modulates protein synthesis via influencing assembly of the translational machinery (56). A newly identified target of mTORC1 La-related protein (LARP1), regulates a specific class of mRNAs (TOP RNAs) including ribosomal mRNAs (RPS and RPL genes) (57–59). These proteins form the 40S and 60S ribosomal subunits critical for protein translation. Upstream regulator analysis of heat-associated DEGs in the placenta associated with HS showed high probability for LARP1-mediated regulation of RPS/RPL proteins. Pathway analysis also indicated coordinated regulation via EIF2 and mTOR/S6K pathways. Importantly, diminished mTOR signaling accentuates heat-induced proteostasis (60) and the UPR inhibits protein synthesis and antagonizes mTORC1 signaling (61). We also observed induction of multiple arms of UPR (ATF6, IRE1, and PERK) in the placenta. Thus, mTOR signaling may serve as a hub integrating nutrient status and response to HS and MNS may mitigate HS via upregulated mTORC1 signaling. Further studies in controlled models of hyperthermia are needed to study these mechanisms in detail in relation to fetal growth.

In addition to its strengths, the present study has some limitations. First, given the retrospective nature of the analysis, individual-level assessment of HS was unavailable. Prospective studies are needed to capture both maternal and fetal HS during pregnancy. We focused on widely used measures of heat exposure such as maximal daily temperature and heat index. More physiologically based thermal exposure measures such as the Universal Thermal Climate Index (UTCI) may be more closely linked to physiological outcomes. However, recent studies have suggested similar performance of UTCI and wet-bulb globe temperature on fetal heart rate (15). The present study focused on cumulative heat exposure over pregnancy trimesters on neonatal outcomes. The impact of acute heat events in specific pregnancy periods were not studied. While the study team carefully assured cold-chain management for biospecimen collection and transport, the direct impact of ambient temperature on samples post collection was not systematically studied. This would not impact the neonatal anthropometric outcomes reported in the paper. While deep transcriptional phenotyping of placenta provided a number of potential mechanisms, placental samples were collected at term. Thus, developmental events in early pregnancy proximate to the heat exposure that contribute to growth retardation need to be investigated through other approaches. Finally, despite the strong associations between heat exposure and poor growth, individual responses may differ depending on other unmeasured variables. Hence, the present study should be considered proof-of-concept.

In conclusion, we present evidence of diminished intrauterine growth associated with greater ambient temperature. The findings highlight the impact of environmental HS on birth length and head circumference and provides additional mechanistic insights into specific pathways associated with diminished fetal growth. Most notably, our findings provide support for improving maternal nutrition as a scalable pathway to improve resilience against HS in austere settings.

## Supplementary Material

pgac309_Supplemental_File

## Data Availability

All data that support the findings of this study are available in this manuscript and the Supplementary Materials. Raw data for transcriptome analysis are available at the Gene Expression Omnibus repository as GSE220877.
