# Correction to: Maternal nutritional status modifies heat-associated growth restriction in women with chronic malnutrition

**DOI:** 10.1093/pnasnexus/pgad368

**Published:** 2023-11-15

This is a correction to: Kartik Shankar, Sumera A Ali, Meghan L Ruebel, Saleem Jessani, Sarah J Borengasser, Stephanie P Gilley, Puujee Jambal, Deaunabah N Yazza, Nicholas Weaver, Jennifer F Kemp, Jamie L Westcott, Audrey E Hendricks, Sarah Saleem, Robert L Goldenberg, K Michael Hambidge, Nancy F Krebs, Maternal nutritional status modifies heat-associated growth restriction in women with chronic malnutrition, *PNAS Nexus*, Volume 2, Issue 1, January 2023, pgac309, https://doi.org/10.1093/pnasnexus/pgac309

During a retroactive audit conducted by *PNAS Nexus*, it was discovered that this paper was missing a statement acknowledging compliance with the *PNAS Nexus* Human and Animal Participants and Clinical Trials policy:

Written informed consent was obtained from all participants prior to enrollment into the study. All study procedures and protocols were approved by the Colorado Multiple Institutional Review Board, University of Colorado, and the local and/or national ethics committees for each of the sites (registered with and the US Office of Human Research Protection and with Federal-wide Assurance in place), and the Data Coordinating Center (RTI International). The study was registered with clinicaltrials.gov (NCT01883193). Additionally, a federally constituted Data Monitoring Committee reviewed the study progress for safety, trial progress, data completion, supplement compliance, and protocol violations twice yearly. All study procedures are also described in detail in a previous publication describing the study protocol (Hambidge KM, et al. 2014., Cited as Ref 18 in the article) and in Hambidge KM et al., 2019 Cited as ref 42; describing the primary outcomes of the trial.

This error has been corrected in the original article.

