# Supplementary material for: Maternal nutritional status modifies heat-associated growth restriction in women with chronic malnutrition
Source: PNAS Nexus. 2023 Jan 27;2(1):pgac309. doi: 10.1093/pnasnexus/pgac309 (PMC9896899; doi:10.1093/pnasnexus/pgac309)
Supplement: pgac309_Supplemental_File [file pgac309_supplemental_file.zip › PNASNEXUS-PNASNEXUS-2022-00669-s02.docx]

**Supplementary Methods**

**Placental gene expression analysis.** Placental tissue was collected from 4 representative locations (1 cm^3^ each), washed, and immersed into PBS with protease and phosphatase inhibitors for protein analysis or RNA*later*^®^ for gene expression analysis (Thermo Fisher Scientific, Waltham, MA, USA). Samples were processed according to manufacturer’s instructions and stored at −80°C until further analysis. Collection time and storage temperature were recorded until samples were shipped to the University of Colorado, Denver, USA. Total RNA was isolated from villous placenta stored in RNA*later*^®^ using a combination of TRI reagent (Molecular Research Center, Cincinnati, OH, USA) and RNeasy-mini columns, including on-column deoxyribonuclease digestion (Qiagen, Germantown, MD USA)(1, 2). Directional cDNA libraries for mRNA-sequencing were prepared using polyA-mRNA from individual RNA samples using Illumina TruSeq reagents (1, 2). Briefly, 1 μg of total RNA was utilized for mRNA isolation using poly-A magnetic beads and was fragmented prior to first and second strand cDNA synthesis. Libraries were generated via PCR including dual-indexed barcodes. Following purification with AmpureXP beads libraries were quantitated using Qubit dsDNA reagents (Invitrogen, Waltham, MA, USA). Pooled cDNA libraries were sequenced (150 bp paired reads) using a NovaSeq 6000 instrument (Illumina, San Diego, CA, USA). Data analysis is described in the statistical analyses section.

**Immunoblotting.** Total placental tissue lysates (n = 12 per group for heat stress and non-HS controls) were prepared in radio-immunoprecipitation assay (RIPA) buffer containing 20 mM Tris-HCl (pH 7.5), 150 mM NaCl, 1 mM EDTA, 1 mM EGTA, 1% NP-40, 1% sodium deoxycholate, 2.5 mM sodium pyrophosphate, 1 mM beta-glycerophosphate, 1 mM sodium orthovanadate and 1 µg/ml leupeptin. Prior to use, RIPA buffer was supplemented with 1 mM PMSF, protease and phosphatase inhibitor cocktails (Halt inhibitors). Samples were lysed using a bead-beating homogenizer (Thermo Scientific) and centrifuged at 10,000 *g* for 10 min at 4ºC to clarify lysates. Total protein concentrations were determined using the BCA assay kit (Pierce™ BCA Protein Assay Kit, Thermo Scientific). Immunoblotting was carried out using the ProteinSimple Wes electrophoresis system that performs size-based capillary electrophoresis and immunodetection in an automated manner (bio-techne, Minneapolis, MN, USA). Protein lysates were prepared at a final concentration of 0.4 mg/mL following Protein Simple Wes manufacturer’s instructions. Size-separated proteins were immobilized within the capillary and probed with antibodies specific for phospho-EIF2α, total EIF2α, phospho p70-S6K, total p70-S6K, phospho 4EBP-1, total 4EBP-1, phospho-AMPK and total AMPK and vinculin (1:1000 dilution, all antibodies were procured from Cell Signalling Technologies Inc., Danvers, MA, USA). Proteins were visualized using labelled rabbit secondary antibodies and quantitated using the Compass software (ProteinSimple). For each lane, densitometric value for each protein was normalized to the value of vinculin control. Data were expressed as ratio of phosphorylated to total levels of each protein.

**Blood metabolite analysis** **using dried blood spot cards**. Non-fasting blood samples were collected by venipuncture at 34 wk gestation (n = 131). Approximately 0.5 mL of whole blood was applied to a Whatman 903 protein saver dried blood spot (DBS) card (GE Healthcare Life Sciences) and dried for at least 4 hours. DBS cards were then stored at −20°C with desiccant packs and humidity indicator cards. A targeted quantitative one-carbon and amino acid analysis panel was performed using LC-MS/MS at the Southeast Center for Integrated Metabolomics (3). The standard panel consisted of 36 amino acids, methylated amino acids and 1C metabolites. From this panel, light-sensitive metabolites S-adenosylmethionine and S-adenosylhomocysteine were excluded as the DBS cards were not specifically protected from light. Briefly, two 3-mm punches from DBS were used for 2 assays to determine the 1C metabolite and AA concentrations. For the 1C–AA assay, blood spots were rehydrated with water and internal standard mix. For the thiol assay, DBSs were rehydrated with water, internal standard mix and BondBreaker (ThermoFisher Scientific). All samples from both assays were then centrifuged and the supernatant was transferred to a 96-well plate for analysis by LC-MS/MS. Samples were separated on a Waters Cortecs HILIC column and eluted with an acetonitrile–water gradient containing ammonium formate and formic acid. Detection was carried on a Bruker EvoQ Elite MS/MS (Bruker, Billerica, MA) in positive ion mode, using a heated electrospray ionization source. For metabolite quantitation, authentic isotopically labelled forms of each metabolite were used as internal standards. Peak area ratios were calculated by dividing the metabolite peak area by the peak area of its internal standard. Metabolite concentrations were calculated by comparing these peak area ratios to standard curves prepared using authentic standards.

References

1. J. Saben *et al.*, Early growth response protein-1 mediates lipotoxicity-associated placental inflammation: role in maternal obesity. *Am J Physiol Endocrinol Metab* **305**, E1-14 (2013).

2. K. Shankar *et al.*, Transcriptomic and epigenomic landscapes during cell fusion in BeWo trophoblast cells. *Placenta* **36**, 1342-1351 (2015).

3. S. P. Gilley *et al.*, Longitudinal Changes of One-Carbon Metabolites and Amino Acid Concentrations during Pregnancy in the Women First Maternal Nutrition Trial. *Curr Dev Nutr* **4**, nzz132 (2020).

**Supplementary Figures**


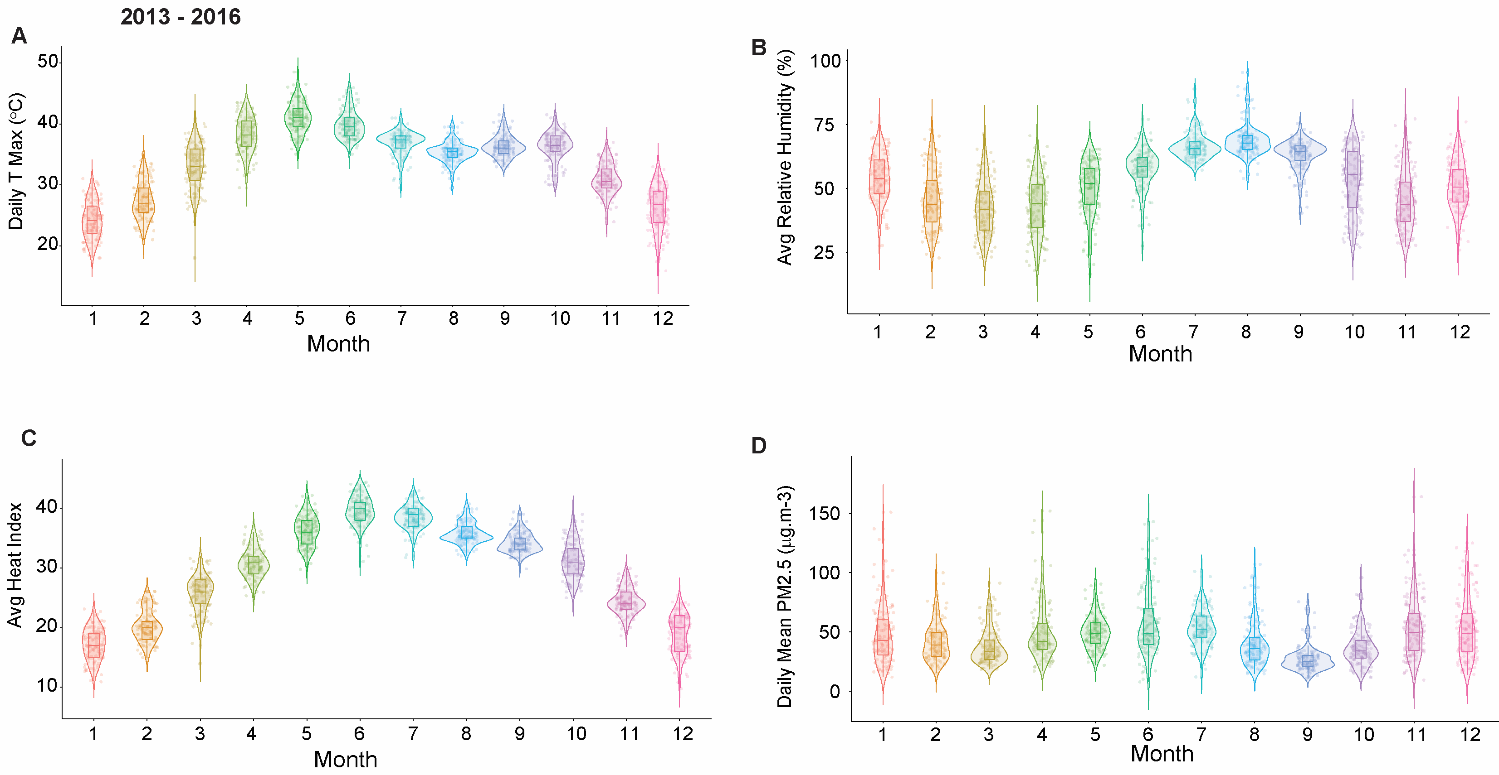


**Figure S1. Daily average values for heat and other environmental variables through the study period.** Violin plots showing distribution of daily average **(A)** maximum air temperature (T_max_) **(B)** Relative humidity, **(C)** Heat index and **(D)** PM2.5 levels from 2013 – 2016 utilized in the analysis. Values are plotted as daily means by month and include between 113 to 122 observations per month.

**
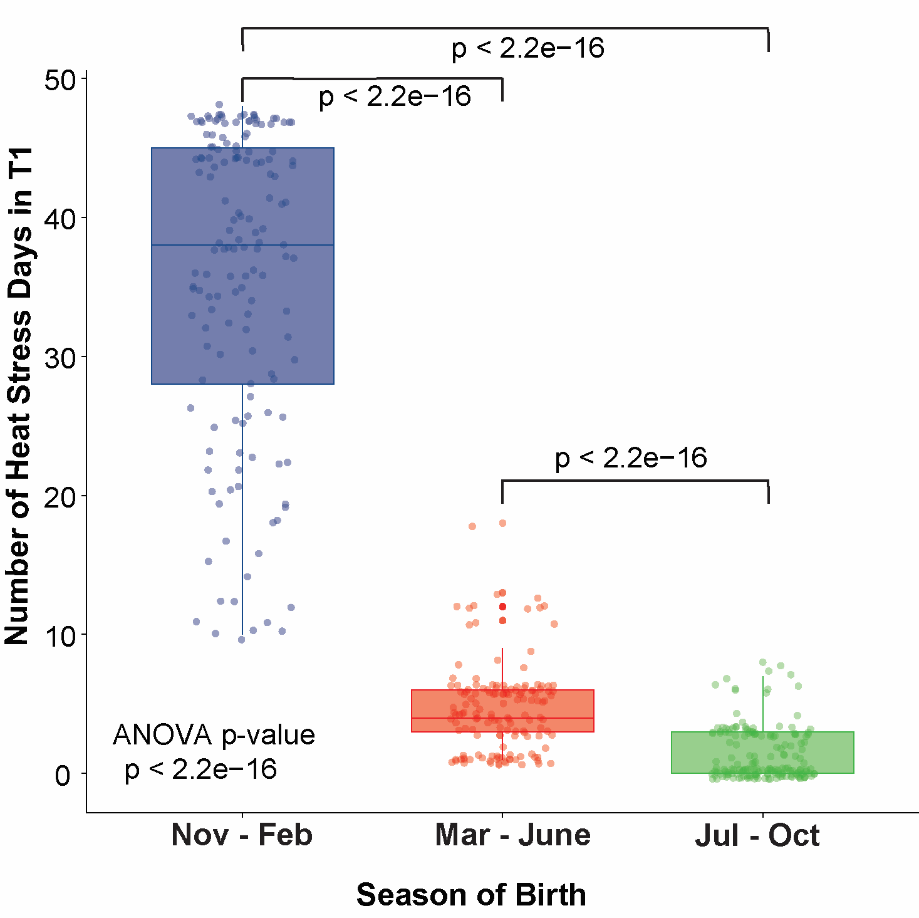
**

**Figure S2. Seasonal variation in heat stress days in the first trimester.** Box plots showing the number of heat stress days in the first trimester based on infant’s season of birth. Nov-Feb is winter (n = 149), Mar – June is summer (n = 143), July – October is rainy season (n = 163), respectively. Heat stress days were defined as days when maximal daily temperature exceeded 39°C. Differences between season were analyzed via one-way ANOVA followed by post-hoc comparisons using Tukey HSD.

**
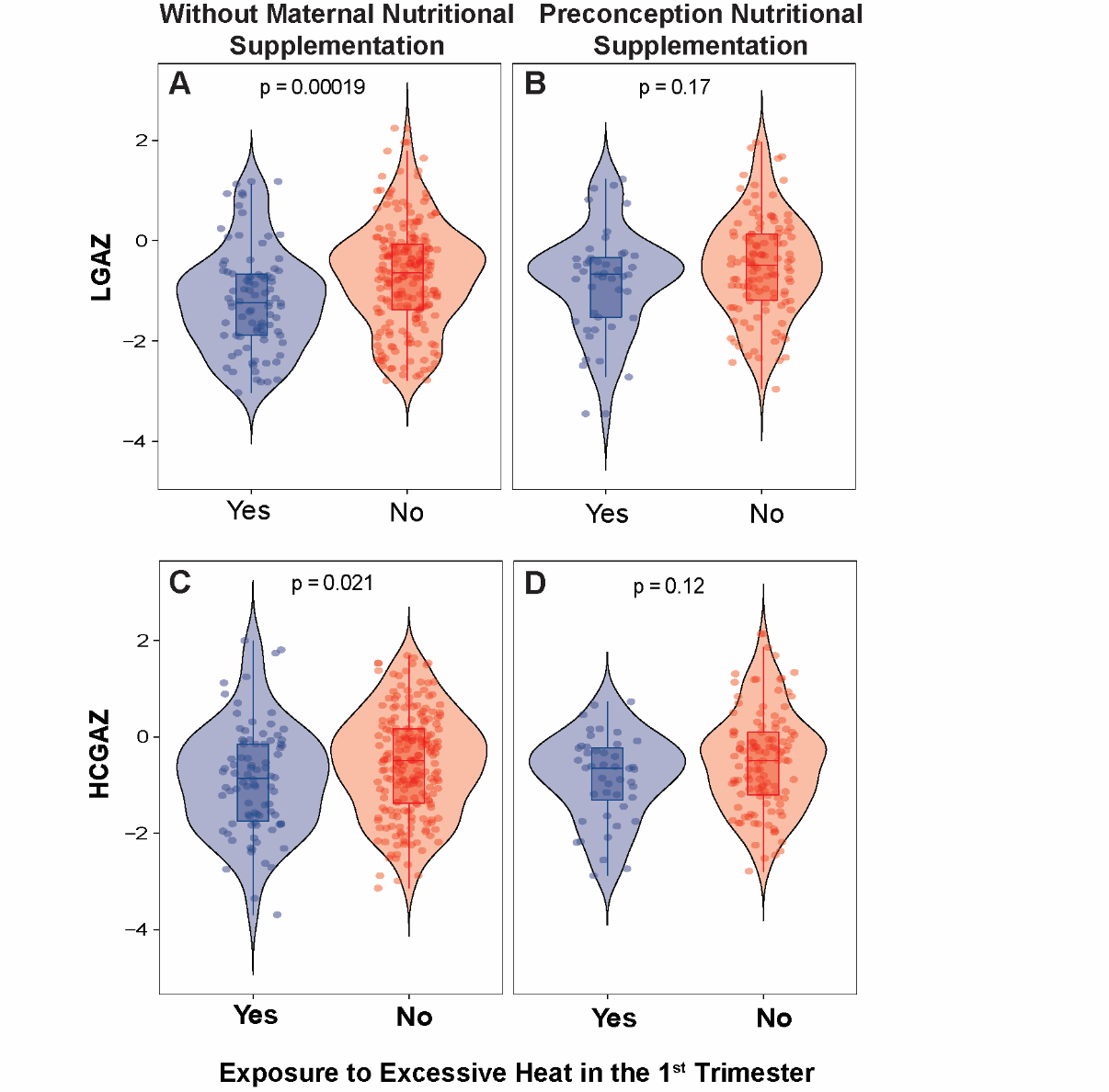
**

**Figure S3. Association between excessive heat exposure and neonatal outcomes stratified by maternal nutritional supplementation.** LGAZ and HCGAZ at birth of infants born to mothers who were either un-supplemented or supplemented with MNS prior to conception. Excessive heat stress was defined as >20 days with T_max_ over 39°C in the first trimester. LGAZ, length-for-age Z-score. Samples sizes were n = 86 and 207 in control and excess HS groups in un-supplemented mothers (left panel, A and C); n = 44 and 114 in the control and excess HS groups among supplemented mothers (right panel, B and D), respectively. P-values were derived using Wilcoxon test. Results of covariate-adjusted linear regression models are presented in Tables.


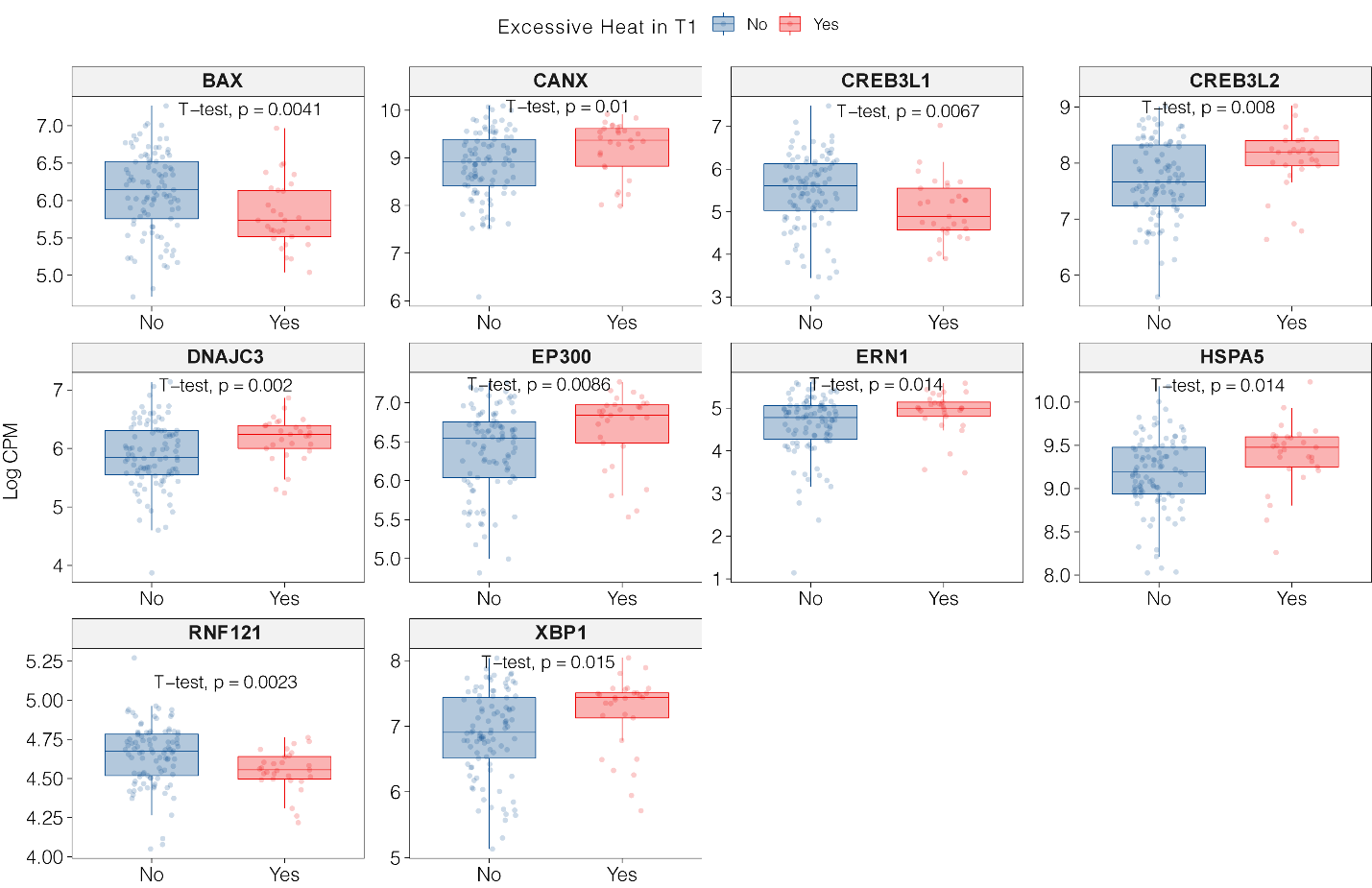


**Figure S4. mRNA expression of gene involved in UPR pathways associated with excessive heat stress.** mRNA expression of gene involved in unfolded protein response (UPR, ER stress) in term placentas (n = 126). Excessive heat stress was defined as >20 days with T_max_ over 39°C in the first trimester. (n= 29 HS; n = 97 non-HS controls). CPM = counts per million reads.


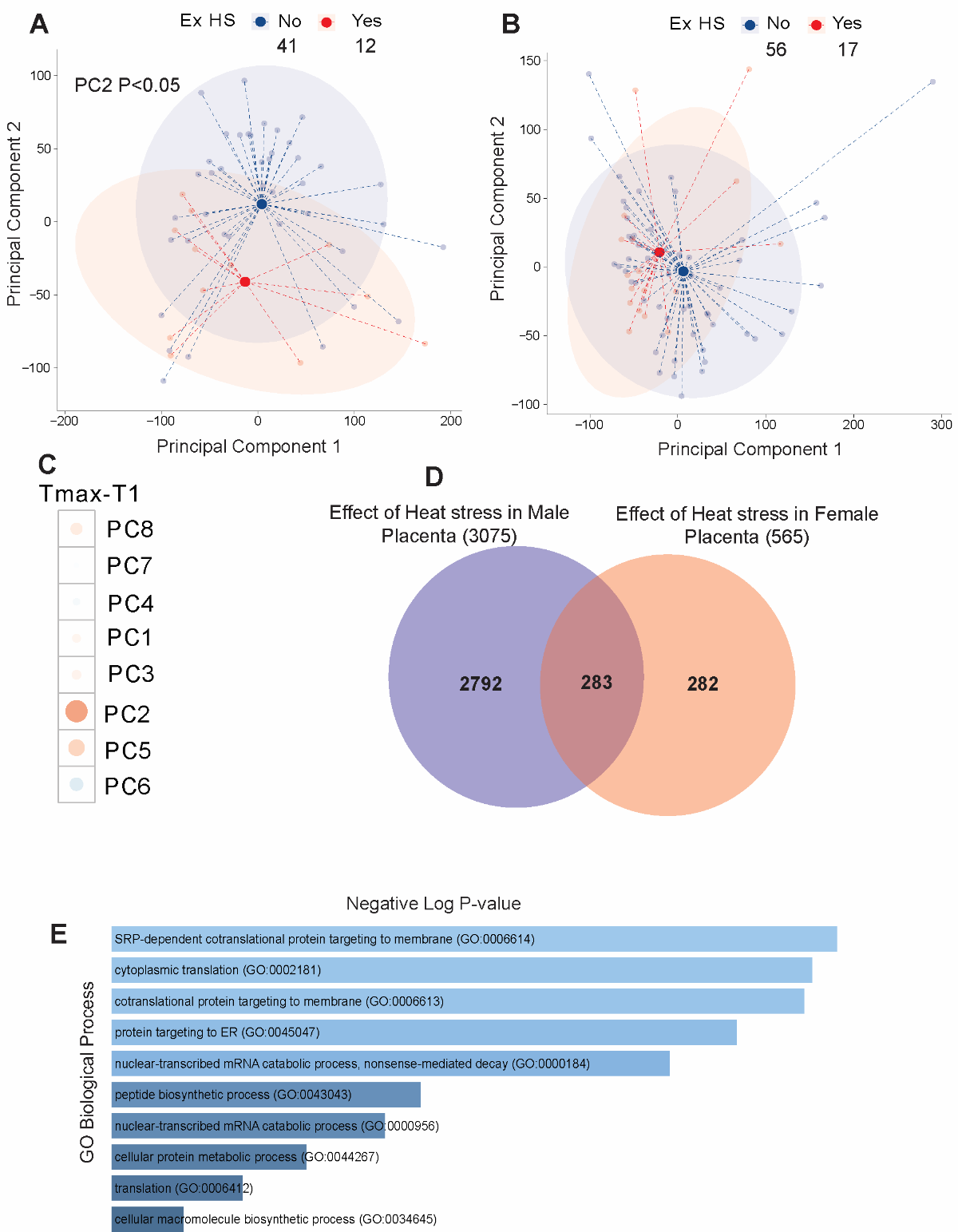


**Figure S5. Sex-stratified analysis of placental gene expression changes associated with excessive heat stress.** Global transcriptomic analysis of term villous placentas was conducted using mRNA-sequencing (n = 126). Data analysis to identify differentially expressed genes (DEGs) was done based on excessive heat stress defined as >20 days with T_max_ over 39°C in the first trimester. Sample sizes in the non-heat stress controls and HS groups were 56 and 17 in females, and 41 and 12 in males, respectively. Principal components analysis of all expressed genes in **(A)** male and **(B)** female placenta showing significant heat-associated effect in males (principal component 2, Student’s t-test P<0.05). **(C)** Associations between PCs and T_max_ in the first trimester showing significant (p = 0.0007) correlation with PC2. **(D)** Venn diagram showing overlap between DEGs altered in male and female placenta. **(E)** GO biological processes enriched among commonly regulated (283 genes) in both sexes showing significant enrichment of protein translation genes.


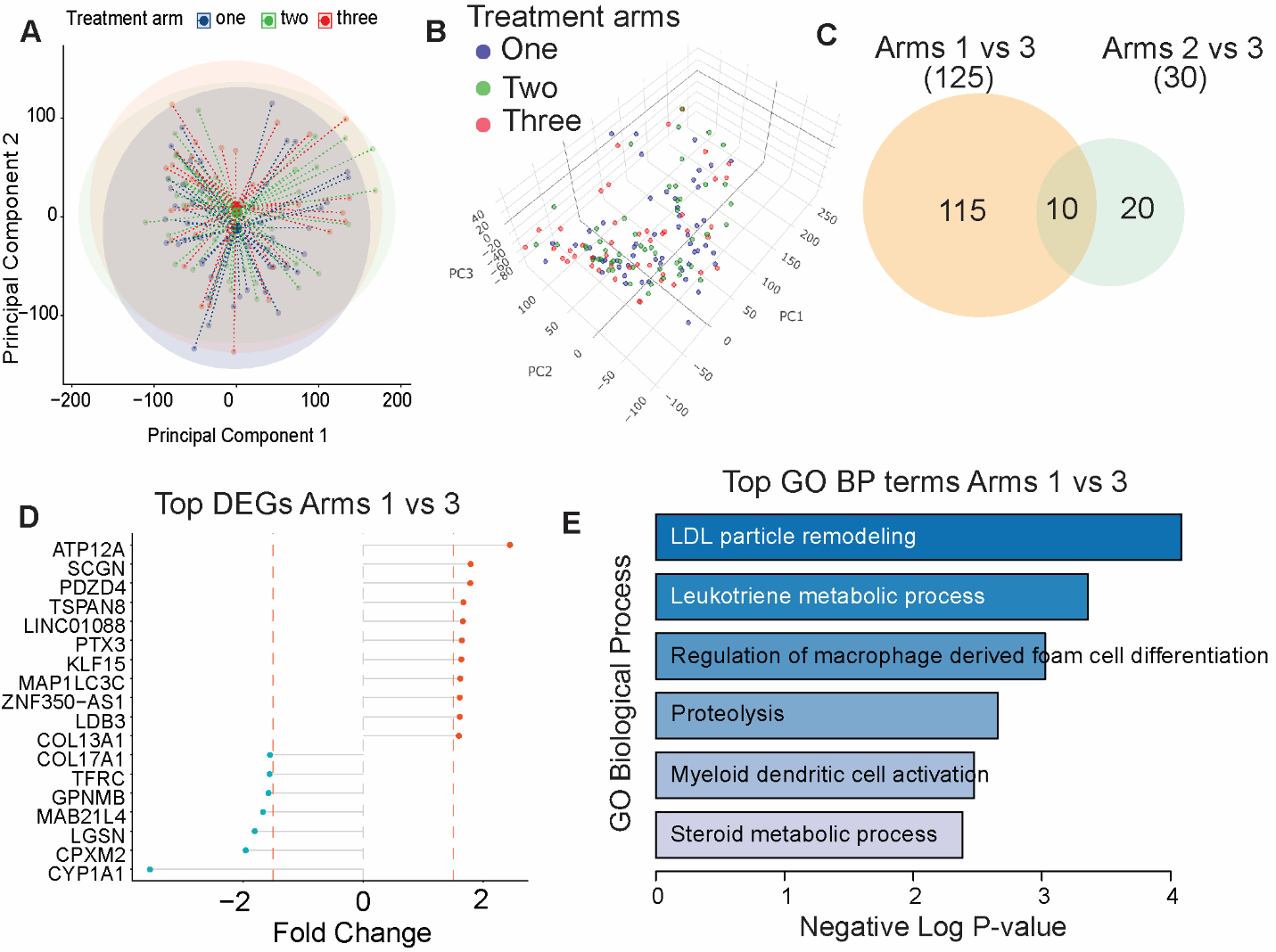


**Figure S6. Effects of MNS supplementation on placental gene expression.** Global transcriptomic analysis of term villous placentas was conducted using mRNA-sequencing (n = 126). Data analysis to identify differentially expressed genes (DEGs) was done based MNS intervention arms. Sample sizes were 47, 42 and 37 in arms 1, 2 and 3, respectively. **(A)** 2D and **(B)** 3D- representation of principal components analysis of all expressed genes showing no major separation between arms. **(C)** Venn diagram showing overlap between DEGs in 1 vs 3 and 2 vs 3 comparisons (p<0.05; ±1.4-fold change). **(D)** Top DEGs altered comparison arms 1 vs 3, representing preconception + gestational MNS. **(E)** GO biological processes enriched among genes altered with preconception + gestational MNS.

**
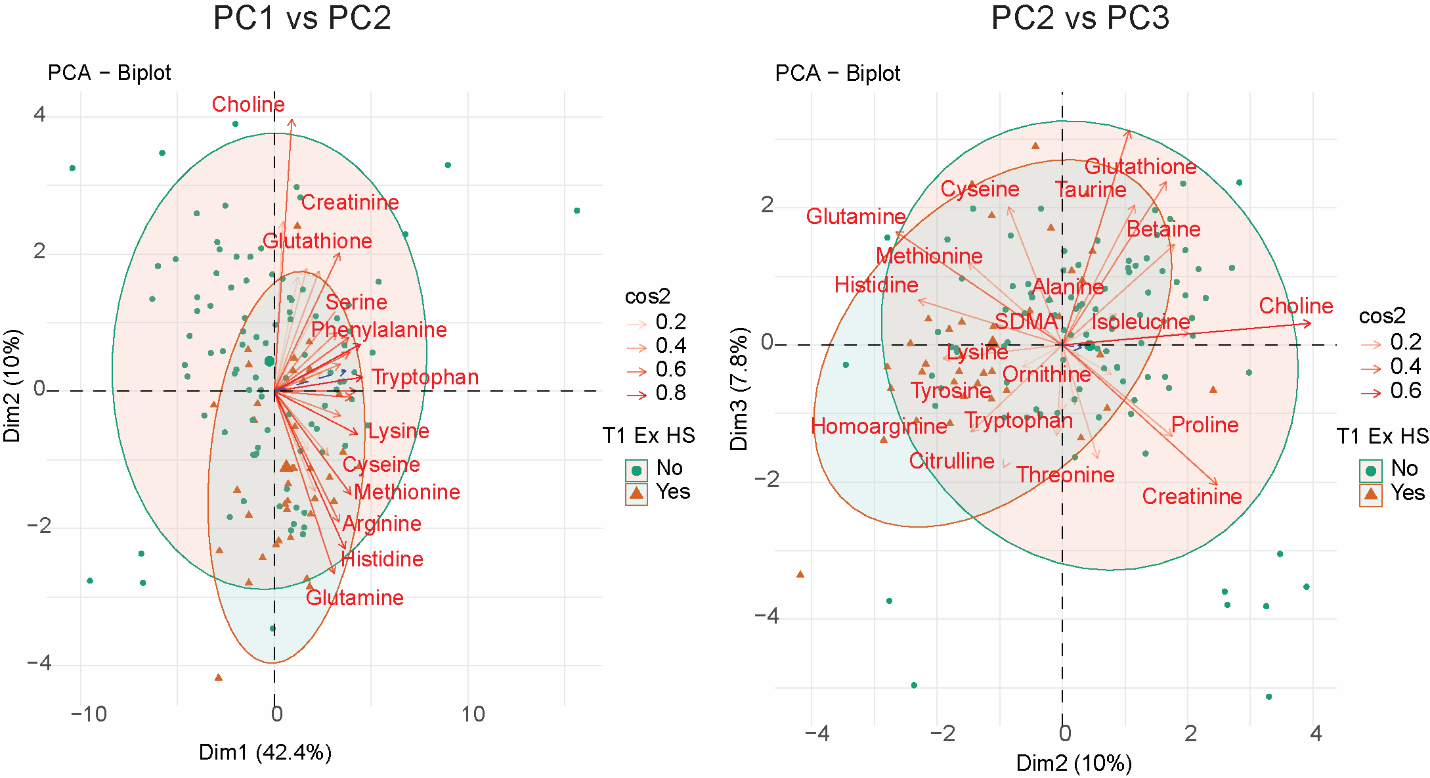
**

**Figure S7. Global changes in targeted metabolite profiles with heat stress.** PCA bi-plots showing samples and metabolites across PC1/PC2 and PC2/PC3 components from non-HS and HS groups. PCA analysis was done using the FactoMineR package. Metabolite profiles included 27 metabolies for which quantitative data was available via LC-MS/MS (total n = 131). Samples sizes were n = 94 in non-HS and 37 in HS groups.
